# Thriving Through Stressful Life Events with Nature: A Mixed-Method Study on Tending Indoor Plants and Rumination Resilience

**DOI:** 10.3390/ijerph22030369

**Published:** 2025-03-03

**Authors:** Samieul Azad, Melissa Marselle

**Affiliations:** Faculty of Health and Medical Sciences, Environmental Psychology, School of Psychology, University of Surrey, Stag Hill Campus, Guildford GU2 7XH, UK; m.marselle@surrey.ac.uk

**Keywords:** stressful life events, indoor plants, rumination, resilience, nature-based intervention

## Abstract

Stressful life events are often undesirable, inevitable, and significant changes in one’s life, often triggering rumination and posing risks to mental health. However, these risks can be managed through coping strategies. Contact with nature has been shown to reduce rumination and enhance mental well-being. The current study investigated the effectiveness of a one-month nature-based intervention in enhancing psychological well-being and building resilience to manage rumination following a stressful life event. In this mixed-method study, 26 participants were randomly allocated to either an experimental group (*n* = 13), which tended to the *Zamioculcas zamiifolia* indoor plant for one month, or a waitlist control group (*n* = 13). Quantitative findings showed that tending to indoor plants was significantly effective in reducing depressive symptoms (*p* = 0.003), perceived stress (*p* < 0.001), negative affect (*p* = 0.017), and rumination (*p* = 0.015), as well as in enhancing resilience (*p* = 0.03) compared to the control group post-intervention. Qualitative findings provided insight into how the nature-based intervention fosters rumination resilience, the mediating effects of tending to an indoor plant, and the contribution it makes to psychological well-being. ‘Offers a slice of nature by bringing the outside, in’ demonstrates how caring for indoor plants creates a bridge for connection with nature. ‘Fosters an emotionally regulating personal sanctuary’ captures how tending to indoor plants can help manage emotions and provide a sense of empowerment that helps mitigate the tendency to ruminate. ‘Plants seeds for improving self-care, personal growth and introspection’ highlights indoor plants as a symbolic representation of resilience and renewal. A narrative emerges: as indoor plants grow and thrive with attention, so too does the individual, forming a deep, reciprocal relationship between nature and personal well-being. This study demonstrates nature’s role in coping with stressful life events and developing rumination resilience, paving the way for further research to explore its caveats and refine and expand nature-based interventions.

## 1. Introduction

### 1.1. Overview

Stressful life events are significant occurrences or changes in one’s life that demand adjustments to people’s typical activities [1]. Often considered undesirable [2], stressful life events are an inevitable part of our lives [3], including experiences such as family conflicts (marital disputes, parent–child disagreements), personal crises (bereavement, relationship breakdowns), financial struggles (unemployment, inability to meet basic needs), and health challenges (chronic illnesses, acute medical conditions) [4,5]. Currently, the United Kingdom (UK) is facing a cost-of-living crisis that is affecting the most financially vulnerable individuals, leading to psychological distress and anxiety [6,7]. Nuffield’s Healthier Nation Index reported that the cost-of-living crisis has affected the physical health of 60% of individuals and the mental health of 59% of individuals [8]. Common aftermaths of exposure to such events include psychological distress (emotional turmoil and cognitive disruption), depression, and anxiety [9]. To reduce mental health risks and build resilience after a stressful life event, effective and accessible solutions are necessary [10]. Effective management through coping strategies and social support can mitigate the negative effects of stressful life events [11]. Among the various coping strategies, nature-based interventions have proven to be particularly effective [12]. The growing interest in nature-based interventions for stressful life events stems from the increasing recognition of the benefits that nature provides in enhancing mental health and well-being. The UK government’s 25-year plan includes several policies aimed at connecting people with the environment to improve health and well-being. One of these policies involves ‘Exploring ways to integrate environmental therapies into mental health services’ [13]. Engaging with nature can help manage the effects of stressful life events and build resilience, especially in addressing a significant consequence of such events: rumination. Contact with nature could be one such solution; studies have shown that contact with nature can facilitate recovery from stress [12,14], reduce rumination [15] and symptoms of depression [16], and foster resilience [17,18]. The current study investigates the effectiveness of a one-month nature-based intervention in enhancing psychological well-being and building resilience to manage rumination following a stressful life event.

### 1.2. Rumination and Psychological Resilience

A major consequence of stressful life events is the onset of rumination, in which individuals engage in persistent and repetitive negative thoughts about the event and its implications [19]. Types of rumination include brooding (feelings of self-criticism and helplessness) and reflective pondering (intentions of understanding and resolving these feelings) [19,20]. It can prevent individuals from moving past these experiences and hinder their ability to engage in positive, constructive activities that promote well-being. In this way, rumination acts as a bridge between stressful life events and the development of symptoms of depression and anxiety, thereby exacerbating the negative impact on mental well-being [19,21]. Ruscio et al. [21] show that greater rumination after a stressful life event predicts worse moods, more maladaptive behaviour, and greater symptoms of major depressive disorder (MDD) and generalised anxiety disorder (GAD). Rumination was found to be more harmful in individuals diagnosed with depression and anxiety and lasted longer in those vulnerable to emotional disorders [21], suggesting that rumination could be a risk factor in stress sensitivity, depression, and anxiety.

Consequently, fostering psychological resilience to manage rumination is essential for mitigating the adverse effects of stressful life events. Psychological resilience is the ability to adapt and recover from adversity, stress, or trauma, enabling individuals to bounce back from setbacks, maintain well-being, and continue functioning effectively despite adverse conditions [22]. To facilitate resilience, protective factors are required. Protective factors help buffer the impact of stress and adversity and enhance our ability to cope [23,24]. Recovery (the ability to return to normal functioning after a significant stressor or adversity) and transformation (recovery accompanied by experiences of positive growth and change) [23] are promoted by protective factors, according to resilience theory characteristics [23]. This study considers the natural environment as a protective factor for recovery and transformative resilience [24,25,26].

Rumination resilience is an individual’s ability/capacity to manage and mitigate the negative effects of rumination. Managing rumination following a stressful life event requires minimising the judgemental form of rumination (brooding) while increasing the purposeful and analytical form of rumination (reflection). This shift from brooding to reflective rumination fosters resilience by promoting both recovery and transformation. By engaging in reflection rather than brooding, individuals reframe their experiences and build a more resilient mindset. In this way, reflective rumination acts as a cognitive mechanism that transforms adversity into an opportunity for growth, making it a key driver of transformative resilience [27,28]. This can be achieved through a complex web of gratifying experiences and a healthy lifestyle [29]. One such gratifying experience this study considers is contact with nature.

### 1.3. Nature Contact and Rumination Resilience

**Figure 1:**
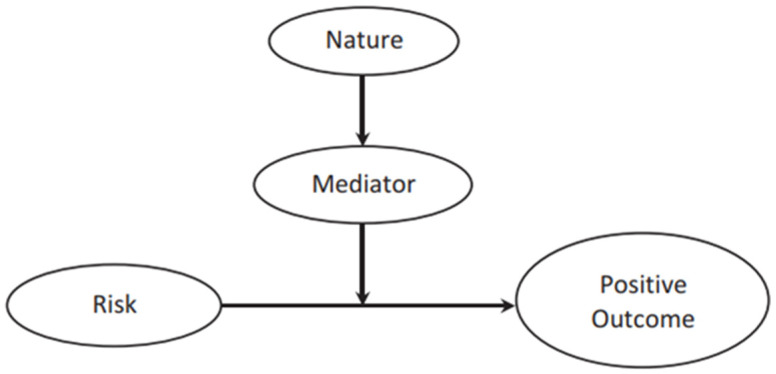
Wells [26] mediated moderator of resilience: mediating pathways that may explain nature’s moderator effect.

Contact with nature has been demonstrated to be a promising approach for supporting individuals in building and maintaining biological, psychological, and social resilience-related resources [30], promoting nature-based biopsychosocial resilience, as contact with nature provides numerous opportunities to build resilience and helps people cope with life’s inevitable challenges. White et al.’s. [30] Nature-Based Biopsychosocial Resilience Theory (NBRT) integrates three perspectives on nature-health relations by outlining how resilience resources (e.g., improved immune function, better emotional regulation, and strengthened social empathy) are deployed across prevention, response, and recovery phases and that nature-based interventions at various levels of health promotion and disease prevention help build and sustain these resilience resources, reinforcing the role of nature in overall well-being. Access to nature and green spaces can reduce health risks, potentially through the underlying mechanisms of improved executive cognitive functions and social connections as mediated pathways (the intermediate steps from nature contact to health improvements) (Figure 1). Figure 1 shows mediated moderation.

Mediated moderation occurs when a mediator explains the process through which a moderator influences the relationship between an independent and dependent variable. While a moderator alters the strength or direction of this relationship (e.g., nature buffering the impact of risk on health outcomes), a mediator helps uncover how or why this moderation occurs. For example, if nature exposure moderates the link between stress and health by reducing negative effects, mediated moderation would examine the underlying mechanism (e.g., emotion regulation) that explains why nature has this buffering effect. It helps identify the process through which the moderated effect is produced, providing deeper insight into resilience-building mechanisms [26]. These mediated pathways hypothesise how/why nature acts as a moderator in the resilience process, serving as a protective factor that facilitates adaptive coping and recovery from stressful events [26].

A handful of studies suggest that engaging with nature could foster resilience and support mental health in the face of stressful life events. Chawla et al. [31] found that engaging students at both elementary and high school levels in green schoolyard activities helped develop key protective factors for resilience, such as feelings of competence and supportive social relationships. Research by Wells and Evans [32] found that children with abundant access to nearby nature exhibit greater resilience to life stressors compared to those with limited exposure, suggesting that nature can serve as a protective buffer. Individuals with more green space within a 3 km radius were less affected by stressful life events, as indicated by lower levels of health complaints and perceived general health (health indicators), than those with less green space, as well as a marginally significant, similar pattern observed in perceived mental health [17]. Similarly, Marselle et al. [18] highlighted the beneficial impact of group walks in nature on mental health and recovery resilience for those experiencing stressful life events, suggesting an ‘undoing’ effect. However, neither walking in nature nor frequent nature walks moderated the impact of stressful life events on mental health.

However, in these studies, the role of active engagement with nature in reducing the persistent adverse effects of rumination and buffering the impact of stressful life events remains underexplored. Active engagement with nature refers to intentional, hands-on interaction with natural environments [33,34] (e.g., gardening and green exercise); an exploration of which can provide coping strategies for integrating nature-based practices into daily life, thereby promoting psychological resilience. This study addresses this gap by investigating how contact with nature can promote rumination resilience and help mitigate these negative effects.

Much of the existing research has also concentrated solely on contact with outdoor nature; therefore, a study that expands the focus to include nature engagement indoors is needed, especially in environments where outdoor access may be limited, considering that we spend a substantial amount of time in indoor settings (e.g., home, work).

These studies may oversimplify the relationship between nature contact as a buffer, resilience, and positive health outcomes, especially given the indefinite mechanism, i.e., the not fully understood processes of how or why nature contact produces these positive effects. As a result, robust methodological approaches can help explore why the moderating effect of nature is not consistently observed [18]. Inconsistencies also include health benefits based on racial or ethnic groups [35] and between environments [33], as well as measures of exposure to nature [12]. This may involve reevaluating how contact with nature is considered a buffer and delving deeper into narratives of resilience that extend beyond self-report measures. Consequently, this study adopts a qualitative approach, along with a quantitative approach, to gain a deeper understanding of the personal, complex, and context-dependent ways in which nature contact serves as a buffer against stressful life events. By integrating statistical evidence with personal narratives, this mixed-method approach advances knowledge in nature contact research.

### 1.4. Restoration and Reflection

Contact with nature can reduce the risk of mental ill health following the experience of a stressful life event by providing adaptive resources—protective factors—that can be utilised to buffer the negative effects of the stressor on mental health, thereby making recovery faster and more complete [26,30]. In this way, nature’s adaptive resources serve as mediators that buffer or moderate the negative effects of stressful life events on mental health [26] (Figure 1). Moderators or protective factors in the resilience process are variables that influence how individuals respond to stress or adversity, helping to enhance their ability to cope and recover. One adaptive resource in this model is directed attention.

Directed attention is an executive cognitive function that enables us to focus, maintain concentration, process information, and plan and solve problems [36,37]. Problem-solving is an essential protective factor for resilience [38]. However, the ability to direct attention requires effort and is thus a limited cognitive resource that can become fatigued with overuse, leading to mental fatigue, increased stress, and irritability, which reduce individuals’ ability to concentrate, make decisions, and perform cognitively [39]—all of which impair our ability to solve problems [39] and deal with stressful events [40]. When this happens, one needs to restore the ability to direct attention. Stressful life events significantly deplete directed attention by increasing cognitive load, potentially causing emotional distress and inducing rumination [41]. Effective stress management and restorative practices, like spending time in nature, are important for mitigating these effects. Attention Restoration Theory (ART) [37] states that contact with nature can restore the ability to direct attention when exposed to an environment that contains four restorative qualities—fascination, being away, extent, and compatibility—that capture effortless attention, allowing for the replenishment of cognitive resources that have been depleted by sustained directed attention following stressful life events [42,43]. People experiencing stressful life events show improvements in directed attention following contact with nature. Kuo [42] found that people living in social housing were experiencing a variety of stressful life events related to poverty (e.g., family conflict, financial struggles, safety). However, those who lived near more nature had significantly greater directed attention and the ability/capacity to plan and navigate life challenges compared to residents who lived in barren environments. Women with breast cancer who engaged in nature-based activities three times per week showed significant improvement in directed attention compared to women in the standard-care control group [44]. Following attention restoration, ART [37] posits that an additional, higher-order benefit of nature contact is reflection, which involves thinking about life matters and reflecting on one’s life, goals, and priorities and how to achieve them [37]. Reflection may assist individuals in processing their thoughts and emotions, fostering insights and personal growth following a stressful life event [18]. The compatibility and expansiveness of the natural environment promote reflection on personal experiences [45], which is considered crucial for psychological resilience [10,28]. Reflective and transcendent experiences in natural settings have been shown to enhance well-being [46,47]. ART [37] provides the backbone for understanding why an increased push for nature-based interventions is warranted, especially to build rumination resilience. This study focuses on ART [37] due to the appropriateness of rumination [15] and supports recovery resilience. These studies emphasise the positive impact of nature on psychological resilience, particularly through attention restoration and reflection. However, they largely neglect the potential benefits of indoor nature interventions, like caring for indoor plants, in achieving similar psychological outcomes. While ART and reflection play key roles in processing emotions and encouraging personal growth after stressful events, there is still a gap in understanding how indoor nature engagement can support these processes, especially for individuals with limited access to outdoor environments or spend a significant amount of time indoors.

### 1.5. Tending to Indoor Plants as a Nature-Based Intervention

Nature-based interventions encompass various therapeutic practices that use natural environments to promote physical, psychological, and emotional well-being, based on ART [37] theory, which posits that such interactions possess restorative aspects [40]. A few examples of these interventions include horticultural therapy, forest bathing (shinrin-yoku), and green exercise [48]. Forest bathing (individuals immersing themselves in the forest environment to reduce stress and enhance overall well-being [49,50,51]), horticultural therapy (the use of gardening and plant-based activities to aid in recovery and rehabilitation, i.e., connecting patients with nature to improve their physical, mental, and emotional health [52,53,54]), and green exercise (physical fitness and activities within the natural environment to reduce mental fatigue, such as walking [48,55]), have demonstrated their effectiveness in promoting improved well-being. However, these interventions often rely on individuals having access to and dedicated time for natural environments, specialised spaces, or specific equipment, which may not be feasible for everyone, particularly those living in urban areas or with limited mobility. As a result, there is a gap in the literature regarding more accessible forms of nature-based interventions. Indoor plants, on the other hand, not only complement these nature-based interventions but also offer a flexible, practical, and convenient approach to integrating nature into everyday life, particularly in urban and indoor environments [56,57].

An indoor plant is any plant that is grown indoors in places such as residences and offices, for example, peace lily, Zanzibar gem, and Echeveria [58]. Indoor plants as a nature-based intervention offer frequent and prolonged contact with nature [59,60] and the opportunity to interact with nature intentionally and directly [61,62], as well as the flexibility (variety and aesthetic) within personal spaces [63] to improve an individual’s psychological and physiological well-being [64,65,66]. Studies have shown that engaging with indoor plants (for example, transplanting houseplants for 15 min) can significantly reduce psychological and physiological stress, offering a respite from the demands of mental work [67]. Additionally, simply gazing at indoor plants has been found to alleviate work-related stress [68], and the presence of indoor plants has been associated with lower blood pressure and improved cognitive function [69]. Interviews have found that medical professionals associated indoor plants and outdoor nature exposure with improved wellness and reduced burnout, including enhanced mood, physical well-being, and cognitive restoration [70]. Watering indoor plants was found to reduce blood pressure, enhance relaxation, and increase feelings of happiness, highlighting the mental and physical health benefits of the simplicity of plant care [71]. Chamola et al. [72] highlighted the benefits of indoor plants for individuals in enclosed spaces during the COVID-19 pandemic (a stressful life event that caused sudden and significant disruptions to daily routines, including restrictions on movement, social isolation, and financial instability), with 86% of respondents (from the Indian population) reporting reduced stress levels, concluding that indoor plants served as a valuable and accessible tool for alleviating mental and physical stress. Ma’s [62] cross-sectional survey confirms that tending to more plants and spending more time on houseplant care are associated with higher levels of mental health well-being and mindfulness, particularly in urban China. Considering the urban environment of many UK cities, indoor plant research within the UK population is needed to validate these mental health benefits and promote nature-based interventions [73].

This study highlights the need for accessible and suitable nature-based interventions, given the UK government’s growing emphasis on integrating environmental therapies into mental health services. Encouraging plant care would be a practical, low-cost intervention to support stress reduction, resilience, and overall psychological well-being. Despite the clear benefits of indoor plants on well-being, no study has investigated the relationship between indoor plants and resilience following a stressful life event. Tending to indoor plants as a nature-based solution into daily life routines offers a practical and meaningful approach to managing and overcoming ruminative thought patterns following stressful life events. By explicitly focusing on the act of plant care, this study contributes to a nuanced understanding of how intentional, small-scale interactions with nature can increase psychological resilience.

In summary, integrating nature-based interventions, particularly indoor plants, shows promise as a strategy for promoting psychological resilience and reducing the negative effects of rumination in response to stressful life events. This study examines the therapeutic benefits of nature contact and indoor plant interactions as a nature-based intervention for coping with stressful life events, testing whether tending to indoor plants serves as a protective factor/buffer against the negative impact of stressful events on well-being. It seeks to determine whether indoor plants can improve mental health outcomes, support resilience, and reduce rumination in adults who have experienced a recent stressful life event. This study is motivated by the increasing recognition of nature-based interventions in mental health care. While existing research highlights the benefits of green spaces, forest bathing, and outdoor gardening, little is known about indoor plant care as a potential nature-based intervention. Considering the challenges of limited outdoor space in urban environments, the ability to cultivate mental well-being through indoor plants presents an accessible and scalable alternative. Additionally, with many individuals spending significantly more time indoors—whether at work or home—there is an increased opportunity to integrate nature into daily life. This study also fills a gap in the literature by investigating how nature interactions aid in coping with stressful life events and evaluating the effectiveness of indoor plant interventions on rumination resilience. This research addresses the critical issue of improving psychological health and well-being while reducing the risks associated with rumination.

### 1.6. Current Study

The current study is mixed-method research that uses both quantitative and qualitative approaches. The purpose of this study was to determine whether tending an indoor plant for one month fosters resilience by maintaining or improving mental health in individuals who are experiencing a recent stressful life event. As a result, this study investigates whether tending to indoor plants encourages rumination resilience for individuals coping with stressful life events. It was hypothesised that participants who tend to an indoor plant for one month will demonstrate lower levels of depressive symptoms, perceived stress, and rumination, along with higher scores of positive affect and resilience compared to a waitlist control group. A secondary hypothesis was that the experimental group would show a significant change in difference in pre- and post-intervention scores, as measured via well-being and mental health psychological scales. Using qualitative semi-structured interviews, this study also aims to understand the experimental group’s experiences regarding their connection between indoor plant care and their rumination resilience journey. To answer the research question, What are participants’ connection experiences between indoor plant care and their rumination resilience journey following a recent stressful life event?

## 2. Materials and Methods

### 2.1. Research Design

An explanatory sequential mixed-method design [74] was used to assess the effectiveness and participant experience of a nature-based intervention (NBI) on rumination resilience for those experiencing a recent stressful life event. The NBI in this study involves tending to an indoor plant for one month. Initially, this study quantifies the intervention’s impact to establish statistical significance. Then, qualitative data is collected to explain participants’ experiences during their indoor plant care. This study adopts a pragmatic epistemological position [75,76,77,78] driven by the research objectives, and that combination of different approaches provides a broader understanding of the phenomena being investigated [79]. It prioritises practical outcomes, methodological flexibility, and the integration of multiple perspectives to provide a thorough and actionable understanding [80] of the nature-based intervention’s impact on mental health and well-being.

To test the effectiveness of the NBI, a randomised waitlist-controlled trial was conducted. Measures of mental health were taken at the start (T1) and at the end of the study one month later (T2). After the first assessment, participants were randomly assigned in Qualtrics to either the experimental group, which received the NBI, or the waitlist control group. To assess experience, follow-up interviews were conducted for the experimental group only.

### 2.2. Participants

A total of 30 people were recruited from the population who had experienced a recent stressful life event and lived in London and Guildford in southern England. To be eligible for this study, participants had to be adults aged 18 and above, live in London or Guildford (to receive the indoor plant), and have experienced at least one stressful life event in the past six months (according to the LTE-Q [81], see Section 2.3). While previous studies on resilience assessed stressful life events in the past three months [17,18], six months was selected to capture a wider sample population; previous research has shown that experiencing stressful events in the past six months poses the greatest risk for mental ill health [82]. The stressful life events occurred close to the timing of the nature-based intervention to better understand its effects on participants’ well-being [82,83,84]. During recruitment, individuals were excluded if they had not experienced at least one stressful life event in the past six months or if they lived outside the London and Guildford areas.

Participants were recruited using convenience sampling (personal contacts of the researcher) and snowball sampling (recommendations via personal contacts, existing participants, or interested individuals). Recruitment occurred through social media channels, online advertising, and community boards, including LinkedIn, Facebook, Instagram, and Reddit, as well as through direct messaging and emails to the researcher’s contacts. No financial compensation was provided; the only incentive was that participants would receive an indoor houseplant, which they could keep.

Out of the 30 people who completed the stressful life events questionnaire, 26 participants were eligible (65% female, age range 20–54). Participants who had recently experienced at least one stressful life event were randomly allocated to either the experimental NBI group (*n* = 13) or a waitlist control group (*n* = 13) via the Qualtrics Randomizer.

All participants completed this study with no dropouts; there were 26 participants in the final sample. The sample size of 26 participants is consistent with other NBI studies [85,86,87]. A study similar to the current study—a six-week wetland NBI for anxiety and depression treatment, using a mixed-methods design—had a sample of 16 participants; significant improvements in mental well-being, anxiety, stress, and emotional well-being were found following the six-week NBI [88].

This is particularly relevant in areas where larger samples may not be feasible due to resource constraints, recruitment challenges, and participant requirements (i.e., having to be interviewed about recent stressful life events [89]). While the sample size is relatively small, random assignment to experimental and control groups enhances internal validity by reducing selection bias [90,91]; however, it is a limitation this study acknowledges.

Thirteen participants in the experimental group took part in follow-up interviews (85% female, ages 20–27). To be eligible for the interviews, participants must have tended to their indoor plants for one month and completed the second questionnaire survey (T2). Table 1 presents key demographic characteristics of the two groups (experimental and control group participants).

### 2.3. Measures

To check for eligibility, potential participants completed the LTE-Q [81], which is a 12-item self-report checklist measuring the occurrence of stressful life events in the past six months. Items include suffering from serious illness, relationship breakup, family loss, unemployment, or financial burdens. Response options for each item were yes or no. The LTE-Q was chosen because it has been used in previous studies assessing whether contact with nature can foster resilience [17,18].

Our dependent variables are psychological scales measuring mental health and psychological well-being outcomes in adults who have experienced a stressful life event. For all scales, participants respond based on time instructions regarding how they felt over the last month.

The Connor–Davidson Resilience Scale (CD-RISC-10) [92] is a self-report measure of participants’ resilience. It comprises 10 items (e.g., not easily discouraged by failure, thinks of self as a strong person), each rated on a 5-point Likert scale (0 = not true at all, 4 = true nearly all of the time). Cumulative scores range from 0 to 40; higher scores indicate greater resilience. The CD-RISC-10 has demonstrated reliability [93,94] and validity, accurately evaluating resilience as being more stable than the 25-item CD-RISC [92].

The Major Depression Inventory (MDI) [95] is a self-report measure to assess the severity of depressive symptoms according to both ICD-10 and DSM-IV criteria [96,97]. It consists of 10 items (e.g., Have you felt less self-confident? and Have you ever felt very restless?) on a 6-point Likert scale (0 = at no time, 5 = all the time). Cumulative scores range from 0 to 50; higher scores indicate severe depression. The MDI has demonstrated strong reliability [97,98] and validity [99,100], proving to be a valuable and effective assessment tool, both as a diagnostic instrument and as a general rating scale for depression [95].

The Positive and Negative Affect Schedule (PANAS-SF) [101] was used to determine participants’ self-reported emotional state. It comprises two 10-item positive (e.g., interested, proud) and negative (e.g., distressed, ashamed) subscales on a 5-point Likert scale (1 = very slightly or not at all, 5 = extremely). For each subscale, cumulative scores range from 10 to 50; higher scores indicate a greater positive or negative affect. It is known for its reliability [102], validity [103,104], brevity, and ease of administration in assessing positive and negative emotions [101].

The Perceived Stress Scale (PSS-10) [105] is a self-report measure designed to assess participants’ frequency of experiencing stressful thoughts and feelings. It consists of 10 items (e.g., How often have you felt that you were on top of things? and How often have you felt nervous and ‘stressed’?) rated on a 5-point Likert scale (0 = never, 4 = very often). Cumulative scores range from 0 to 40; higher scores indicate greater psychological stress. It is widely used due to its reliability and validity in assessing perceived stress [106,107]. Lee’s [107] systematic literature review found that the 10-item PSS displayed stronger psychometric properties compared to the 14-item version. Validated through studies worldwide, the 10-item Perceived Stress Scale (PSS-10) has become a widely recognised and reliable tool for assessing stress appraisal [108,109,110].

The Ruminative Response Scale (RRS) [111,112] is a self-report measure for levels of ruminative thoughts. It consists of 22 items on a 4-point Likert scale (1 = almost never, 4 = almost always), assessing how often individuals engage in rumination in response to distress or sadness. Cumulative scores range from 22 to 88; higher scores indicate higher degrees of ruminative symptoms. There are three subscales: reflection (purposeful rationalisation) (e.g., writing down what you are thinking about and analysing it), brooding (passive comparison) (e.g., thinking ‘What am I doing to deserve this?’), and depression-related thoughts (e.g., thinking about how alone you feel [111,112,113]). Previous studies have shown that the RRS achieves high reliability [114], proving that it measures an individual’s tendency for ruminative response stably [115] and possesses satisfactory construct and predictive validity [116,117].

#### Covariates

To understand our participants and any influencing factors, both demographic information and connection to nature were measured as covariates. Demographic information included the participant’s age, gender, current residence, and occupation. An individual’s connection to the natural environment has been shown to have an impact on well-being [37], rumination [118], and resilience [119]. Connection to nature was measured using the Nature-Relatedness Scale, NR-6 [120], a 6-item scale (e.g., I take notice of wildlife wherever I am, I always think about how my actions affect the environment) based on a 5-point Likert scale (1 = Disagree, 5 = Agree Strongly) encompassing individual self, perspective, and experience with nature [120]. Cumulative scores range from 6 to 30; higher scores indicate stronger connectedness with the natural environment.

### 2.4. Procedure

This study was approved by the University of Surrey’s University Ethics Committee (FHMS 23-24 160 EGA) (see Supplementary Materials—Supplementary S1).

**Figure 2:**
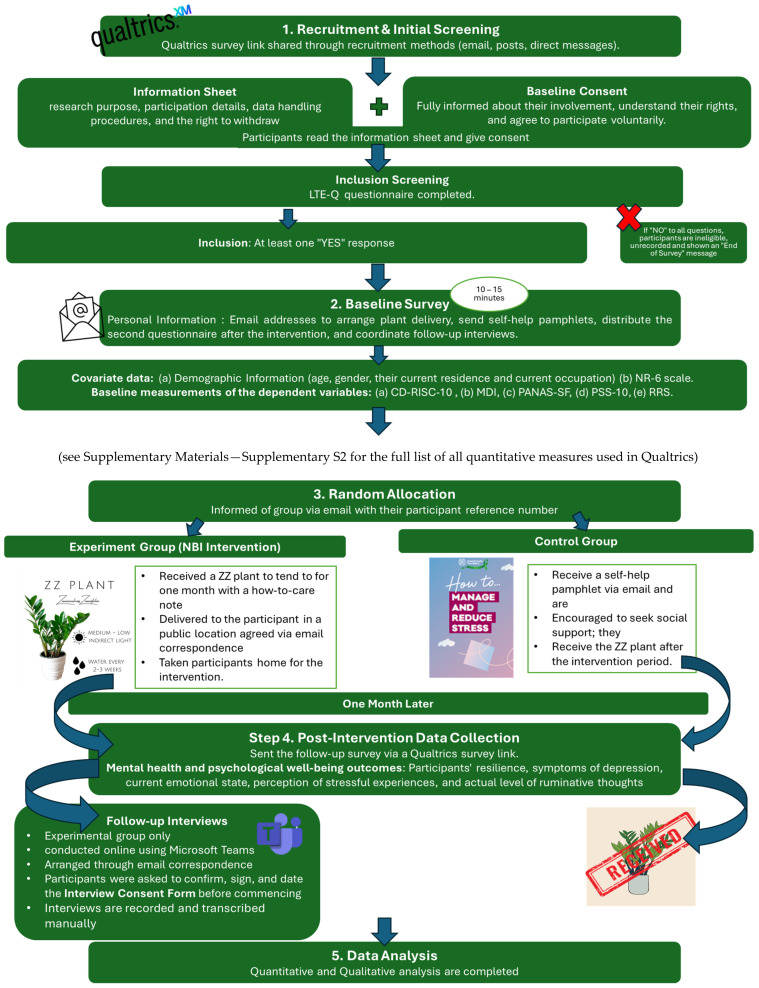
Study design and participant flow.

All quantitative data were collected using an online survey (Qualtrics). Figure 2 shows the study design and participant flow through the study.

Potential participants were provided with an anonymised weblink to the baseline (T1) survey on Qualtrics through recruitment posts, email, and direct messages. After giving informed consent, participants were assessed for eligibility regarding recent stressful life events by completing the LTE-Q [81]. Participants who responded ‘yes’ that they have experienced at least one stressful life event on the LTE-Q in the past six months were included in this study. Potential participants who selected ‘no’ for all LTE-Q items were removed from the survey and debriefed on why they were ineligible for this study. Participants who met the inclusion criteria then progressed through the baseline (T1) survey. Participants were asked for their email addresses to distribute the T2 questionnaire after the intervention. They then provided demographic information, completed the NR-6 [120] scale, and completed the baseline measurements of the dependent variables in the following order: (a) CD-RISC-10 [92], (b) MDI [95], (c) PANAS-SF [101], (d) PSS-10 [105], and (e) RRS [111,112] (Figure 2). The baseline survey took approximately 10–15 min to complete. After completing the baseline survey, participants were randomly allocated to conditions.

The experimental NBI group involved tending to a *Zamioculcas zamiifolia* (ZZ) indoor plant in the residential home for a one-month duration. ZZ plants were chosen due to their low maintenance, adaptability to various indoor conditions, aesthetic appeal, and symbolic representation of resilience [121,122]. The NBI was conducted in participants’ UK homes, where they were responsible for tending to a ZZ plant. Participants were given the plant with a how-to-care note attached (see Supplementary Materials—Supplementary S5). The plant was hand-delivered to the participant at a public location agreed upon through email correspondence and then taken to the participant’s home for the duration of the intervention, which involved tending to a ZZ indoor plant for one month or being part of the waitlist control group.

The waitlist control group was given a self-help pamphlet (Supplementary Materials—Supplementary S6) and encouraged to seek social support [123,124]. Self-help pamphlets and encouragement were given to the control group, as this is a common treatment [125]. Participants in the waitlist control group received the indoor plant (NBI) one month after the T2 survey to ensure ethical considerations (not withholding it entirely so participants are not disadvantaged) and to maintain participant engagement and retention. This waitlist control design allowed for a comparison between groups while ensuring that all participants eventually had access to the potential benefits of the intervention.

One month later, all participants were contacted again and sent the follow-up survey (T2) via a Qualtrics survey link (Figure 2). This survey focused solely on the dependent variables of mental health and psychological well-being outcomes (CD-RISC-10, MDI, PANAS-SF, PSS-10, RRS). All participants kept their plants at the end of this study.

Qualitative data were collected through thirteen individual semi-structured interviews with participants in the experimental group only (Figure 2). All interviews took place after the T2 survey, were conducted by one researcher (SA), and were conducted online using Microsoft Teams. Before starting the individual interview, participants signed the Interview Consent Form, which was obtained via email or in person. The interview did not ask about stressful life events, and participants were told that they could decline to answer any questions if they felt uncomfortable. An interview schedule (Supplementary Materials—Supplementary S3) was created, which consisted of open-ended questions and respective prompts broadly focusing on the NBI, followed by participants’ reflections on the experience, exploring the connection between tending to the plant and their rumination resilience journey. The interviews started by asking participants to describe tending to the ZZ plants (whether they did or not and how), followed by questions about their emotional and psychological experience of the intervention (noticing any changes) and rumination and, lastly, questions exploring the intervention’s broader context and effects (resilience and the natural environment), along with participants’ future intentions to assess the sustained impact of the nature-based intervention. Interviews lasted approximately 15–20 min.

### 2.5. Analysis

All quantitative analyses were performed using Jamovi (version 2.4.11.0, Jamovi Project: Sydney, Australia). Reverse scoring for PSS-10 [105] items 4, 5, 7 and 8 was transformed. To determine whether tending to indoor plants encourages rumination resilience in individuals coping with stressful life events, independent sample *t*-tests were performed on all post-intervention (T2) dependent variable results. This analysis also aimed to determine a significant mean difference between the intervention and control groups after one month. All assumptions for conducting independent *t*-tests were met for all dependent variables, including normality and homogeneity of variance. A mixed factorial 2 × 2 (group × time) ANOVA comparing the dependent variables of both the experimental and control groups over time (T1 and T2) was conducted to determine if the experimental group exhibited greater mental health and well-being than the waitlist control group. The between-subjects factor was the two groups, and the time base served as the within-subjects factor. The assumption of sphericity was met for time. Considering the connection with the nature covariate, an ANCOVA was also conducted to test the hypothesis regarding whether the covariate affected the statistical difference between the experimental and control groups.

All interviews were audio-recorded, anonymised, and transcribed verbatim by one researcher (SA). The interview transcripts were analysed using an inductive approach that included both semantic and latent analysis through reflexive thematic analysis [126]. This method is particularly accessible for novice researchers to examine and analyse qualitative data to construct a narrative about meaningful patterns or themes across the interviews that capture the richness of the research topic. This approach encourages self-reflection on how one’s position and perspective influence the coding and theme development, often leading to a more fluid and flexible coding process [126] (Supplementary Materials—Supplementary S8). Familiarity with the data was gained by continuously re-reading transcripts and generating initial codes, (manually through highlighting text and writing codes in the margins) (see Supplementary Materials—Supplementary S4). Initial codes formed clusters based on similarities. These clusters identified the main themes and subthemes, which were reviewed and defined to create the thematic map (Figure 4) and a narrative that answers the research question and supplements the quantitative data.

## 3. Results

### 3.1. Quantitative Findings

#### 3.1.1. Psychological Well-Being Outcomes at Post-Intervention

This study hypothesised that participants who engaged in tending to indoor plants as a one-month nature-based intervention would demonstrate lower levels of depressive symptoms, perceived stress, negative affect, and rumination, along with higher scores of positive affect and resilience compared to the control group. Table 2 shows the descriptive statistics of the research participant groups at T1 and T2 for all dependent variables.

**Figure 3:**
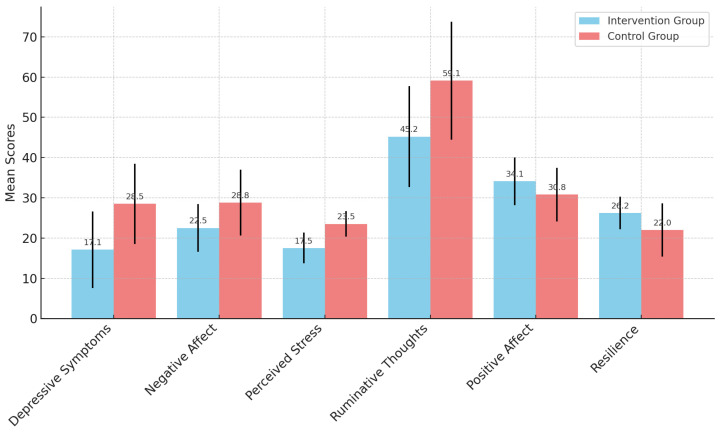
Post-intervention mean scores across dependent variables.

An independent sample *t*-test was performed for all six dependent measures at post-intervention (T2) to identify a significant mean difference between the intervention and control groups after one month. Figure 3 shows the post-intervention means across all the dependent variables for the intervention and control groups, with error bars representing the standard deviation for the experimental group.

The post-intervention level results show that the experimental group (M = 17.1, SD = 9.49, *n* = 13) had significantly lower levels of depressive symptoms at T2 than the control group (M = 28.5, SD = 9.97, *n* = 13): t (24) = 2.98, *p* = 0.003, d = 1.17.

The experimental group (M = 22.5, SD = 5.94, *n* = 13) had significantly lower levels of negative affect at T2 than the control group (M = 28.8, SD = 8.17, *n* = 13): t (24) = 2.25, *p* = 0.017, d = 0.883.

The experimental group (M = 17.5, SD = 3.82, *n* = 13) had significantly lower levels of perceived stress at T2 than the control group (M = 23.5, SD = 3.15, *n* = 13): t (24) = 4.37, *p* = < 0.001, d = 1.713.

The experimental group (M = 45.2, SD = 12.52, *n* = 13) had significantly lower levels of ruminative thoughts at T2 than the control group (M = 59.1, SD = 14.64, *n* = 13): t (23) = 2.31, *p* = 0.015, d = 1.022.

The experimental group (M = 34.1, SD = 5.96, *n* = 13) had higher scores of positive affect at T2 compared to the control group (M = 30.8, SD = 6.67, *n* = 13), although the difference was not statistically significant: t (24) = 1.33, *p* = 0.098, d = 0.523.

The experimental group (M = 26.2, SD = 4.04, *n* = 13) had significantly higher resiliency scores at T2 than the control group (M = 22, SD = 6.66, *n* = 13): t (24) = 1.92, *p* = 0.03, d = 0.754.

Overall, the post-intervention results suggest that tending to plants improved psychological well-being, demonstrating a significant reduction in depression, negative affect, perceived stress, and ruminative thoughts, as well as enhancing resilience post-intervention.

#### 3.1.2. Group Differences in Pre–Post Intervention Scores

A mixed factorial ANOVA was conducted to determine the main effects of groups (experimental and waitlist control) and time (T1 and T2) on the six dependent variables. Time, but not the experimental group, had a significant main effect on mental well-being following the intervention. However, there was a statistically significant interaction between time and experimental conditions. There was a significant main effect of time ((F [1,24] = 30.19), *p* < 0.001, $\eta_{P}^{2}$= 0.56) and dependent variables (depressive symptoms, negative affect, perceived stressed, ruminative thoughts) at post-intervention (M = 29.6, SD = 5.30) that was lower than that at pre-intervention (M = 32.8, SD = 6.02). There was not a significant main effect of the experimental group; participants in the experimental group (M = 29.3, SD = 7.70) did not show a significant difference when compared to the control group (M = 33.1, SD = 7.70) (F [1,24] = 3.14): *p* = 0.089, $\eta_{P}^{2}$ = 0.12. However, time and the experimental group interacted (F [1.24] = 13.83): *p* = 0.047, $\eta_{P}^{2}$= 0.154. This means that there was a main effect of condition and time on all six dependent variables (Table 3), indicating that the experimental group had a larger improvement in mental health, rumination, and resilience compared to the control group.

Overall, the data showed a positive change in mental health between the intervention and control groups over the course of the one-month intervention, suggesting that the nature-based intervention may have had specific beneficial effects.

#### 3.1.3. Covariate

We also tested the nature-relatedness total score (NR-total) as a covariate using ANCOVA and found that there was an insignificant main effect of NR-total (F [1,23] = 0.179): *p* = 0.676, $\eta_{P}^{2}$= 0.008. There was also no statistically significant interaction effect of NR-total on the dependent variables (F [5,115] = 1.474): *p* < 0.204, $\eta_{P}^{2}$= 0.06. This indicates a very small effect size, suggesting that NR-total explains very little variance in the dependent variables; the participants’ level of nature-relatedness (i.e., connection to nature) did not play a significant role in the outcomes of the nature-based intervention. Table 4 shows no visible pattern in the data for nature-relatedness across all dependent variables.The dots (data points) for both conditions are scattered randomly without a clear relationship and the flat regression lines for all dependent variables show the lack of a pattern.

### 3.2. Qualitative Findings

The qualitative findings complement the quantitative findings by explaining why the one-month nature-based intervention positively affected participants’ rumination resilience journey. When asked about the ZZ plant’s current condition, all 13 participants in the experimental group reported that their ZZ plants are alive, healthy, and growing.

**Figure 4:**
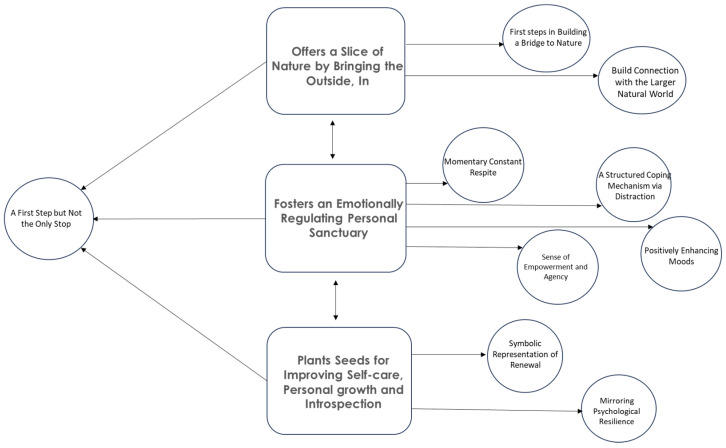
Final thematic map showing three main themes and their respective subthemes.

Three main themes emerged from the thematic analysis (Figure 4): ‘Offers a slice of nature by bringing the outside, in’, ‘Fosters an emotionally regulating personal sanctuary’, and ‘Plants seeds for improving self-care, personal growth and introspection’. These themes illustrate that indoor plants serve as more than just decorative elements; they are vital tools that create a reassuring environment, helping to reduce rumination and build resilience.

Each of the three main themes has respective subthemes, summarised in Table 5, with a description as a key, suggesting a progression from physical interaction with nature to emotional regulation, culminating in personal development, where indoor plants contribute to psychological well-being. All main themes share a common sub-theme—a first step but not the only stop—revealing that although incorporating indoor plants can be an initial beneficial move towards improving well-being, they are not a complete or standalone solution for coping following a stressful life event; there are caveats.

#### 3.2.1. Offers a Slice of Nature by Bringing the Outside in

The majority of participants’ interviews expressed how indoor plants bring the outside natural world inside their homes, often opening the door to step outside to interact with the natural world.

##### Build Connection with the Larger Natural World

Participants expressed that indoor house plants replicate the benefits of nature, suggesting that indoor plants can offer a rewarding, stress-relieving experience within the comfort of home. This is particularly important when it is not possible to go outside or have access to the outdoors;, which is described as follows:

‘be in nature for a bit, you can’t always do that. … having sort of slice of that in the home … makes things feel a bit cosier and a bit stress-free’.

(002)

Similarly, another participant shares how indoor plants provided a positive alternative in a restricted environment as follows:

‘It’s just nice to have. Like a plant in my house … I don’t have a garden or a balcony’.

(006)

Other participants also describe the following:

‘looking after a plant, you kind of realise how fragile it was … you’re in nature … more of an appreciation for … when you see it thriving … of its own accord … you know how much care it takes to just look after this one little plant’.

(002)

This shows how caring for a small piece of nature, like an indoor plant, can enhance one’s understanding and appreciation of the broader natural world, cultivating a greater sense of respect and admiration for the environment.

This intimate connection and admiration for nature shift our perception, as indoor plants are often reminders, which is expressed as follows:

‘having an indoor plant can help you to reconnect with everything that matters … tend to take nature for granted … important to recognise … we need to do to protect the environment … we didn’t get this far without nature, so we need to give it back as much as we took’.

(015)

This highlights how the presence of indoor plants can inspire reflection on our relationship with nature, reinforcing our appreciation and responsibility to care for the environment. We re-evaluate our relationship with the natural environment as it is a duty of care in a reciprocated relationship.

One participant shares their feeling of connection by instinctively protecting their plant and demonstrates how, over time, bonds with nature are not merely decorative; they are something to be cherished and worth nurturing, as follows:

‘when my cat was like trying to eat the plant … I became very protective over the plant so I was no … developed the connection to it over time’.

(015)

##### First Steps in Building a Bridge to Nature

For those who spend much of their time indoors due to caregiving or other commitments, plants become an accessible form of nature. One participant shares that their current life demands make spending time in nature a privilege as follows:

‘looking after my parents and being their caregiver, has meant that I’m kind of stuck inside a lot … plant is sort of like a bridge towards being with nature properly’.

(016)

Additionally, sentiments are shared about how indoor plants often open the door to engage with the outside world as follows:

‘when I was repotting it, I was kind of looking out the window at this big Willow tree … kind of wholesome and at peace … I’m bonding with nature … there was a thunderstorm … I’m dealing with nature, but I’m still inside in the safe, cosy warmth’.

(013)

‘Nice way to like to interact indirectly with kind of like the nature … It’s kind of like meeting it halfway and then it’s trying to get you to do something outside today … do stuff and break the cycle of being isolated’.

(004)

They both highlight how indoor plants foster a connection to nature from the comfort of indoors, gradually encouraging more direct engagement with the outside world. This illustrates the bridge that indoor plants ask you to cross to connect with nature and enhance your well-being.

Overall, this theme reveals how indoor plants act as a crucial intermediary for connecting with nature within the confines of our homes. We gain a bit of the natural environment to form deeper connections, encouraging a more mindful and appreciative attitude toward nature.

##### A First Step, but Not the Only Stop

Although tending to indoor plants has caveats; we must recognise that this nature-based intervention does not always develop a connection with nature. Some participants felt it was limited or insufficient, expressing the following:

‘connection to nature … for me … is more than just that the plants that I have in my house’.

(006)

‘I’m still a bit preoccupied. So I haven’t noticed. Nature quite so much’.

(014)

They also expressed a longing for a more immersive natural experience as follows:

‘I’ve only got the one plant in London … I don’t feel it’s really made me feel that connected to nature … I’d wanna escape more to the countryside’.

(021)

The extent of this connection can depend on personal engagements, the number of plants, and the presence of a few plants indoors, which is not replicable in a more expansive natural environment experience. This shows that indoor plants are steps towards a solution, but not solely the solution; the nature-based intervention is dependent on other factors.

#### 3.2.2. Fosters an Emotionally Regulating Personal Sanctuary

Participants’ experiences provided insights into how tending to indoor plants affected their ruminative thoughts, referring to the space that tending to indoor plants creates: a personal sanctuary. This personal sanctuary supports well-being by providing a personal retreat where individuals can build structure, reflect, and find relief and emotional stability.

##### Positively Enhancing Moods

**Figure 5:**
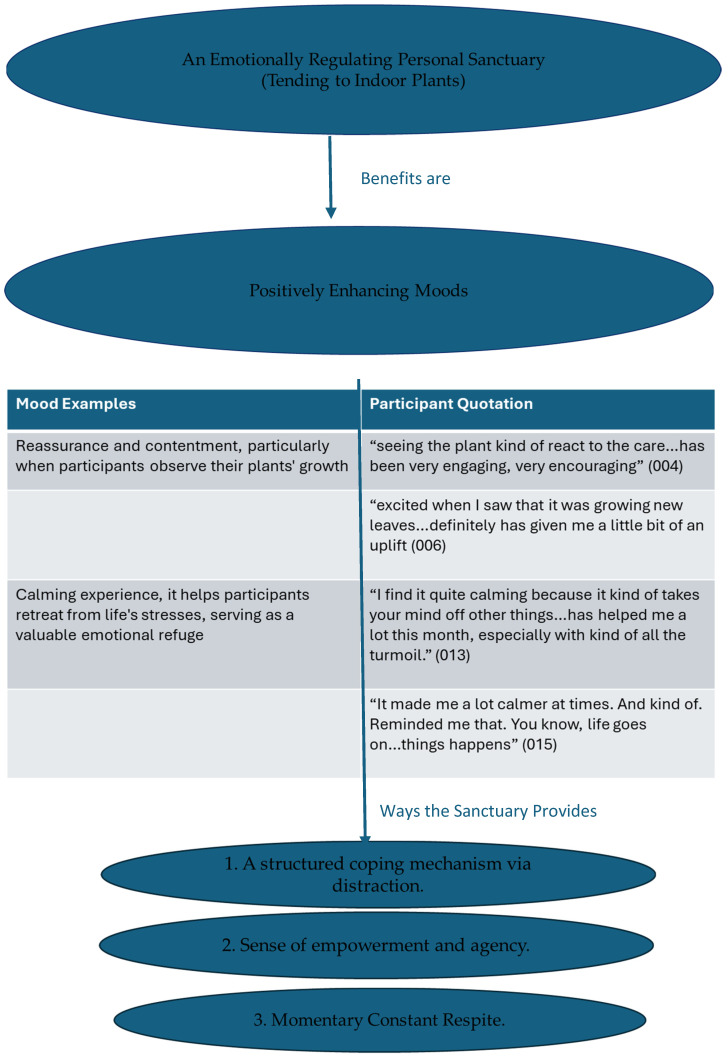
Visual representation of the benefits of and several ways to create a personal sanctuary, i.e., tending to indoor plants.

This study recognises the significance of this personal sanctuary, as participants’ experiences highlight the positive emotional benefits of tending to indoor plants. In evaluating how indoor plants help reduce ruminative thoughts, participants identified several ways this sanctuary provides such benefits. Figure 5 shows how the personal sanctuary leads participants to positively enhancing their moods in three key ways.

##### A Structured Coping Mechanism via Distraction

One way the sanctuary provides is by creating a structured coping mechanism through tending to indoor plants, as it serves as a distraction, focusing our attention away from negative repetitive thoughts.

One participant shares how tending to the indoor plant distracted their minds from the negative repetitive thoughts they had, showing a practical way to break away from those thoughts, as follows:

‘A chore to distract your brain from repetitive thoughts … I think depending on the type of thoughts, I might need a bigger plant … bigger plant, bigger responsibility, bigger distraction’.

(005)

Another participant highlights an interruption to the cycle of rumination, acting in opposition to idleness, providing a meaningful and helpful distraction that contributes to improved mood and behaviour by tending to the indoor plant, as follows:

‘whole distraction of look at me, I’m doing something that is being appreciated by another living being … having something to kind of interrupt that cycle and make me change my behaviour or my mood pattern is really, really important’.

(013)

However, as a meaningful distraction, tending to indoor plants is limited. Even though everyday work pressures become more manageable, deeper emotional challenges, such as grief, are harder to resolve, which is expressed as follows:

‘It distracted me. But I’ve still got the same stresses in life … had a couple of deaths in my family … those are more difficult to get away’.

(021)

This shows that its effectiveness as a distraction may vary depending on the nature and intensity of the stress or emotional challenges being faced.

##### Sense of Empowerment and Agency

Another way this personal sanctuary provides positive benefits is through the sense of control and responsibility that participants experience while tending to the plants.

‘bit of responsibility … keeping something alive … puts things into perspective … makes your problems in your life feel a little bit less. Serious … kind of a base and build from that … you’re looking after the plant … I’ve sorted out this thing … move on to other things rather than just getting swamped by life’.

(002)

This participant shows a grounding sense of responsibility that provides a sense of focus and purpose, helping to manage stress, gain perspective, and serve as a foundation for tackling life’s challenges in a more structured and less overwhelming way.

The relationship between responsibility and control is evident; taking responsibility often leads to a greater sense of control, and feeling in control helps manage responsibilities more effectively.

There is often comfort and empowerment in meeting a plant’s needs, which rewarded by restoring a sense of control and personal agency, particularly during times when other areas of life are uncontrollable. This is expressed as follows:

‘I’ve really been feeling kind of a lack of control over kind of my life and my situation. And so it was really nice to kind of feel that I had something that was just mine that I could control’.

(013)

Another participant similarly shares times when they have felt like they had no control as follows:

‘I’ve been struggling a lot recently with dysphoria … bit of a source of depression and anxiety … comfort in like tending to a plant because you know how to look after it … sometimes it’s hard to keep on top of … everything … but this is something that is manageable and you can kind of start there…move on and see what else we can do’.

(002)

This shows the importance of having something within your ability to control amidst the difficulty of trying to manage the uncontrollable. The predictability of tending to the plant offers comfort and confidence. The confidence gained from this manageable control helps to gradually address other life demands.

Participants have shown that a personal sanctuary offers both meaningful responsibility and a manageable sense of control. The empowerment and agency participants gain from the personal sanctuary provide a practical and accessible way to cope with life’s challenges.

##### Momentary Constant Respite

A final way is the offer of a consistent break—a constant in their life that provides relaxation and relief. Tending to the plant teaches us to take that moment to relax, showing that it is this consistent act that helps people assess their situation with calm and intentionality, breaking cycles of repetitive thinking, expressed as follows:

‘Whenever I took the time to take care of the plant it made me like stop and think and, chill out … be applied to … repetitive thinking … And then think of where you’re going to go next from there … it helps you to slow down a little bit’.

(001)

Furthermore, another participant shows that a personal sanctuary offers a necessary pause to prevent burnout, allowing for a mental reset and reducing feelings of overwhelm, as follows:

‘it just made me feel quite calm in the moment … nice to take a moment out to sort of out of a work routine … If getting stuck with those repetitive thoughts. Which sometimes can like help work through problems’.

(022)

This momentary break is a positive constant, which is expressed as follows:

‘It’s structure I struggle without. Structure when I’m overwhelmed … something persistent in the future is looking after the plant’.

(014)

This shows the personal sanctuary as a non-pressuring presence in their life, providing an emotional safety net and highlighting the benefits of having a constant that is not contingent but aligns with one’s ruminative journey, expressed as follows:

‘it being quite a hardy plant … But you have that kind of delay period that if you are in a bad place, it is something you can come back to … less dependence on you’.

(004)

These participants show that the constancy of the personal sanctuary provides structure, stability, and emotional support, especially with the inconsistency of life’s demands.

Overall, this theme revealed that tending to indoor plants serves as a personal sanctuary, as participants shared the positive emotional benefits of this sanctuary, including meaningful distractions, a sense of control and responsibility, and a consistent break, all of which contributed to reducing rumination. However, the effectiveness of this sanctuary varies depending on the nature and intensity of stressful life events in comparison to other positive factors. This personal sanctuary can be overshadowed by more significant life events, expressed as follows:

‘like my work life. If that’s going well, that’s probably going to have more of an effect on my mood than like looking after the plant’.

(006)

We learn to recognise that this emotionally regulating personal sanctuary is one of many on our journey to coping after a stressful life event.

#### 3.2.3. Plants Seeds for Improving Self-Care, Personal Growth, and Introspection

The primary message of this theme is the participants’ transition towards growth, understanding tending to plants as a symbolic representation of renewal that mirrors our psychological resilience. Through the participants’ experiences, we learn the lessons that tending to plants teaches regarding personal growth and overcoming challenges.

##### Symbolic Representation of Renewal

A participant’s experience reveals the parallel between mistreatment and forgiveness for both themselves and the indoor plant as follows:

‘the initial like neglect reflected some of the neglect of myself that has been going on for a while … good analogy for us as people, isn’t it …’

‘… then I also viewed things like that as a positive in a sense, because sometimes the thing that’s dying off is not always a negative thing … sometimes is growth … maybe that leaf came off ‘cause, it was already tired and then it was. Waiting for other bits to grow … good representation of how I’ve been feeling over the last few months …’

‘… probably shed a leaf in the whole last six months of up and down mental health … a resilience in staying this long on this slightly doomed planet … this plant, all it has to do is just grow … probably same kind of journey I want to take now … just focusing on the things that feel more innate as opposed to all the things that we pile on as a society’.

(017)

This shows the relationship between their journey, mental health, and growth in relation to tending the plant, connecting the neglect of the indoor plant to their self-neglect, implying that their mental and emotional state is mirrored in their ability to care. It shows the power of recognising the symbolic nature of tending to the plant; for instance, the shedding of a leaf symbolises the speaker’s process of shedding what no longer serves them, allowing for new growth and renewal. It also shows the profound connection between one’s inner world and subsequent actions, using the care of a plant as a powerful analogy for personal growth, resilience, and the importance of focusing on what truly matters.

##### Mirroring Psychological Resilience

The act of caring for a plant serves as a metaphor for self-care and personal growth, helping the individual build resilience by validating their ability to nurture life. An important nuance is that just as plants need care and attention to truly thrive, people also need kindness and support, even when they feel resilient, which is expressed as follows:

‘plants are pretty resilient … go through a lot in nature … thrive despite it all … still needing a lot of tending to … interesting way to look resilient … You can thrive through adversity … But, sometimes you do need to be kind to yourself’.

(002)

Resiliency does not mean neglecting self-care; rather, it requires a balance between enduring hardship and allowing oneself the compassion and rest needed to flourish.

How we tend to the plant mirrors how we represent ourselves; it is an extension of the self and symbolises the importance of persistence and the ability to cope with daily stressors and stressful life events, which is expressed as follows:

‘the plant signifies how you are in your daily life as well, I guess coping, and I think how you are in daily life shows how you deal with stressors, daily or eventful’.

(021)

As another participant shares the importance of not giving up as follows:

‘It’s more case of like seeing what works and what doesn’t … kind of not giving up … over watering is the biggest reason why plants die … your kind of tending to stuff isn’t bad just means … reassess the way you’re approaching it’.

(018)

This shows that when an indoor plant does not thrive under particular care, the solution is not to abandon it but to try different approaches. The belief that ‘I can’t keep a plant alive’ (018) is a mindset rather than an actual capability, reflecting resiliency when self-imposed limitations are challenged. It teaches that psychological resilience involves continuous reassessment and a willingness to improve rather than simply pushing forward blindly.

This theme highlights how tending to indoor plants serves as a powerful metaphor for personal growth, self-reflection, and self-care. Participants’ experiences show that caring for plants mirrors their psychological journeys, emphasising self-awareness. This practice offers insights into internal struggles and the impact of actions/inactions, ultimately fostering resilience and well-being.

##### A First Step, but Not the Only Stop

This is not always straightforward insight, and the nuances of life may require more than tending to an indoor plant. Its effectiveness might vary based on the individual’s context, expressed as follows:

‘It’s a good way to act as a steppingstone … a useful tool … like the foundations. Of building resilience … even though I didn’t like to observe it myself’.

(001)

This participant recognises the intervention’s impact without having felt it themselves.

‘I’ve ended up being quite resilient. But the plant has still died, whereas now sort of, I feel like I’ve not been dealing with things as well … but the plants come out of it great … perhaps that’s because before I needed less of a distraction’.

(013)

This suggests that the effects of tending to indoor plants are minimal because they vary based on individual differences. Participants’ experiences reveal the complexity of the relationship between plant care and psychological resilience, emphasising that resilience is multifaceted and depends on personal interpretation.

‘useful in building my resilience in a way, it not exactly the sort of be all and end all … having a purpose helps with the relationship between resilience and tending to indoor plants’.

(016)

Tending to indoor plants is not the quintessential solution for building resilience; rather, it is one stop among many that individuals make on their journey towards rumination resilience.

Overall, these three themes reveal participants’ experiences and the connection between tending to indoor plants and the rumination resilience journey. The narrative that emerges is that tending to indoor plants serves as a multifaceted initial tool for emotional and psychological well-being. It is the moments that take us away from the chaos of stressful life events when we retreat to our personal sanctuary of relief to find a tangible bridge connecting us to nature and learn to nurture ourselves. Participants illustrate how the simple act of tending to indoor plants can have profound and lasting effects on personal growth and emotional well-being, showing that this nature-based intervention is effective in reducing ruminative thoughts and increasing resilience following a stressful life event. However, it is important to recognise that before everyone rushes to their nearest garden centre, tending to indoor plants is not a definitive answer for coping after a stressful life event; it is contextual and a valuable part of this mix.

## 4. Discussion

### 4.1. Summary of Quantitative and Qualitative Results

Quantitative and qualitative findings provide a comprehensive understanding of the impact of tending to indoor plants as a nature-based intervention for enhancing resilience and reducing rumination following stressful life events. The quantitative findings statistically support the main hypothesis and partially support the secondary hypothesis. From this, we can conclude that while the nature-based intervention was effective in reducing depressive symptoms, perceived stress, negative affect, and rumination, as well as in enhancing resilience, it did not significantly affect positive affect or produce a significant overall change in well-being improvement compared to the control group. However, the results suggest that tending to indoor plants can be a promising approach for targeting certain aspects of psychological well-being, particularly in reducing rumination and increasing resilience. Also, nature-relatedness as a covariate did not significantly affect the dependent variables. The qualitative findings identified several themes from individual interviews that describe and answer participants’ experiences regarding their connection between indoor plant care and their rumination resilience journey following a recent stressful life event. Themes include ‘Offers a slice of nature by bringing the outside, in’, ‘Fosters an emotionally regulating personal sanctuary’, and ‘Plants seeds for improving self-care, personal growth and introspection’.

Together, the findings suggest that while the statistical data highlight the intervention’s specific effects on psychological outcomes (depressive symptoms, perceived stress, negative affect, rumination, and resilience), the qualitative data explain how and why these effects occur from the participants’ perspectives, revealing how tending to plants can serve as an emotional anchor and a practical tool for coping following a stressful life event. The combined findings suggest that tending to indoor plants is a valuable, though not exhaustive, approach to enhancing rumination resilience and well-being following a stressful life event.

### 4.2. Interpretation

In line with the hypothesis and purpose of this study, the quantitative data statistically support the nature-based intervention’s effectiveness, while the qualitative findings highlight its nuanced role in the broader journey of emotional and psychological recovery. These findings contribute to the growing body of evidence that nature-based interventions can effectively enhance psychological well-being and fill the gap in terms of effectiveness, specifically in reducing rumination and enhancing resilience after stressful life events. Regarding dependent variables, similar to this study, which demonstrated reduced levels of perceived stress [127], findings reveal that patients in a waiting room exposed to real plants reported significantly lower stress levels compared to a no-plants control condition. Previous research has shown that engaging with indoor plants demonstrates encouraging results in reducing stress and improving wellness [62,69]. Lee et al.’s [67] findings indicate that active interaction with indoor plants can reduce physiological and psychological stress by suppressing sympathetic nervous system activity and lowering diastolic blood pressure. Female participants exhibited improved task performance when a plant was present in the room [128]; this enhancement was found to be attributed to a positive change in mood [128,129]. Reduced depressive and anxiety symptoms were also associated with the abundance of greenery visible from the home and with an increasing number of indoor plants when students felt isolated during the COVID-19 pandemic [130]. This study built on that foundation by having participants engage with the plants and reveal the personal reasoning behind these effects.

Regarding the themes from the thematic analysis, from our participants’ experiences, we understand that tending to plants as a personal sanctuary underscores the importance of creating spaces and moments of respite in our lives. It illustrates how small, intentional actions can offer relief from the chaos of stressful life events, providing a tangible, manageable way to reflect and reconnect with oneself and the natural world, and it mirrors the process of self-care and self-nurturing. These findings are similar to those of a study [131] on veterans with post-traumatic stress disorder and forest therapy garden. These qualitative findings revealed that veterans felt protected enough in nature to close their eyes and experienced feelings of security, illustrating the unchangeable endurance of nature that accepts us just the way we are. This reinforces the idea that simple, meaningful activities can profoundly affect personal growth and emotional well-being [132], which is a theme that is evident in this study, demonstrating that indoor plants foster improvements in our personal growth and introspection. It highlights the potential of accessible, low-cost interventions that can be easily integrated into daily life, particularly in environments where access to outdoor nature is limited [133]. The theme of having a part of outside nature inside is similar to the themes in [134]. Their study found that university students chose study rooms with indoor plants due to a sense of homeliness, along with the relaxing and refreshing environment, qualities [134] that are often associated with spending time in nature [12] and were enhanced by the comfort of a familiar indoor setting.

This study’s findings also emphasise that while tending to indoor plants can be a valuable tool for coping following a stressful life event, it is not a one-size-fits-all approach; its effectiveness is influenced by individual preferences, the novelty of life situations, and the specific challenges we face. It highlights recognising a nature-based intervention for its value in certain situations (e.g., tending to indoor plants as a manageable way to control overwhelming thoughts) but cautions against relying on it as the sole solution for every situation. O’Brien. [135] highlights the flexible, adaptable, and inclusive nature-based programmes that are essential for effectively supporting vulnerable individuals with mental health needs.

### 4.3. Implications

Theoretically, the findings fit with the Attention Restoration Theory [37] to a certain extent. Tending to indoor plants can be seen as engaging in soft fascination [68], which allows the mind to rest from the demands of directed attention, thereby aiding in mental fatigue recovery. Participants experienced tending to indoor plants as a personal sanctuary that provided a space to reflect and a tangible bridge to the natural environment, in line with ART’s [37] position of spending time in nature as a restorative environment. However, these findings also challenge ART [37] in terms of the universality of nature’s restorative effects and its propriety qualities (fascination, being away, extent, and compatibility) for achieving a restorative effect [136], considering the effectiveness of tending to indoor plants in increasing recovery resilience and reducing rumination. It highlights a more nuanced understanding of restoration; our intentional engagement with nature reflects our understanding of its significance [137]. Rather than feeling the need to escape to the outdoors, we incorporate a slice of nature into the comfort and safety of our homes, especially while we struggle with rumination. It is also essential to recognise that tending to indoor plants should be a voluntary, enjoyable activity (not a chore, as indoor plant care is sometimes considered) so as not to diminish its positive impact [138,139].

These findings extend the research conducted by [17,18] regarding coping strategies following stressful life events. It is notably encouraging to observe how beneficial tending to indoor plants has proven to be in the aftermath of such events. While indoor plant care may not be a panacea, it emerges as a valuable supplementary approach alongside more established tools, such as social support, counselling, and other therapeutic interventions [124,140]. The key takeaway is that while no single solution can address all the negative impacts of a stressful life event, recognising and incorporating various tools, such as engaging with nature, can offer additional support.

Practically, the findings suggest that tending to indoor plants can be a valuable recommendation for individuals dealing with significant mental health challenges stemming from stressful life events [71]. Integrating indoor plants into workplace and educational settings could provide colleagues and peers with a tangible way to alleviate stress and foster a calming environment [141]. By introducing indoor plants into our homes, we gain not just added greenery but also an enhancement to our well-being [142]. Gifting plants to those closest to us who are coping with stressful life events has evident positive impacts.

### 4.4. Limitations

The current study acknowledges the use of opportunity sampling, in which the researchers had some level of familiarity with the participants. This familiarity might have inadvertently introduced biases, as participants could have been motivated by their interest in the nature of the intervention or by a desire to perform well for the researcher. Participants might have overstated their positive experiences due to a lack of care for the indoor plants and concerns about appearing ideal, potentially overshadowing the actual impact on their well-being. As participant 017 shared, ‘the worry of judgement wasn’t even about towards the plant … am I going to be useful enough to have this interview’.

Another limitation is that the intervention lasted for only one month. This raises questions about whether the intervention period was sufficient to observe any significant long-term effects. A longer study might provide a clearer understanding of the sustained impact of indoor plant care on rumination and resilience. Additionally, it prompts consideration of how such an intervention would be prescribed to individuals outside this study.

This study also faced limitations related to its sample size and demographic composition. With a small sample size, the majority being female, and the age differences between groups, the generalizability of the findings is limited. Participants in the experimental group were younger than those in the control group, potentially influencing results (i.e., differences in willingness to share during interviews, their alternative coping methods, and life responsibilities). This raises questions about whether age and gender differences play a role in how individuals respond to nature-based interventions like indoor plant care. This study serves as a foundation for future research aiming to replicate the findings with larger and more diverse representative samples.

### 4.5. Future Research

The identification of caveats and the first study using indoor plant care as a nature-based intervention opens up valuable avenues for future research. Future research could explore factors such as the types of plants used, the optimal number of plants, and the duration of the intervention. As participant 013 highlighted, ‘you just have one plant … depending on the type … the benefit could be minimal … my friends has 6 million house plants … fine line … burden rather than an enjoyment’. Future research could employ a longitudinal study that investigates changes in psychological well-being and mental health outcomes after six months and after a year and identifies the optimal time needed to tend to the indoor plant, if any, similar to the study [143] involving two women participating in weekly NBIs on a farm for 3 months. Additionally, there is potential to examine the effects of combining indoor plant care with other nature-based activities, such as gardening, nature walks, or nature photography. By establishing a structured schedule that incorporates these activities, future studies could identify the most effective combinations and practices (green prescription [144]) for enhancing the benefits of nature-based interventions. This approach not only broadens our understanding but also helps tailor interventions to maximise their impact on well-being and individuals’ rumination resilience journeys following a stressful life event.

## 5. Conclusions

To conclude, this study has demonstrated that tending to indoor plants is an effective tool in an individual’s journey towards rumination resilience following a stressful life. The combination of quantitative and qualitative findings provides a comprehensive understanding and nuanced interpretation of the impact of indoor plant care on psychological well-being. These approaches complement each other by bridging the gap between measurable outcomes and personal experiences of tending to indoor plants. The quantitative results highlight the measurable psychological improvements (e.g., in depression and stress) that can be achieved through this simple yet impactful activity. The qualitative findings revealed three key themes that encapsulate participants’ experiences and highlight the deeper impact of indoor plant care on their self-awareness and personal development. This highlights the potential for future research to focus on adapting nature-based interventions as a sanctuary that complements the comfort and security of home to provide additional support for mitigating the effects of stressful life events, particularly in addressing mental health and enhancing coping mechanisms.

## Figures and Tables

**Table 1:** Demographic characteristics of the study sample.

Characteristics	Total Sample (N = 26)	Experimental (*n* = 13)	Control (*n* = 13)
Age (yrs)			
Mean (SD)	26.7(6.75)	24.2(2.17)	29.2(8.77)
(min–max)	20–54	20–27	20–54
Gender			
Male (%)	30.7	7.7	53.8
Female (%)	65.5	84.6	46.2
Other (%)	3.8	7.7	0
Current Residence (City)			
London (%)	65.4	61.5	69.3
Guildford (%)	11.5	15.4	7.6
Other (%)	23.1	23.1	23.1
Occupation (Industry)			
Retail and Hospitality (%)	23.1	23.1	23.1
Tourism and Customer Service (%)	11.5	15.4	7.7
Education and Research (%)	23.1	30.8	15.4
Healthcare and Science (%)	7.7	0	15.4
Environmental and Sustainability (%)	3.8	7.7	0
Administration and Office Work (%)	15.4	15.4	15.4
Self-Employment (%)	3.8	0	7.7
Unemployed (%)	11.5	7.7	15.4

**Table 2:** Descriptive statistics and mean scores of all measures in each group.

		Groups (N = 26)			
		Experimental (*n* = 13)		Control (*n* = 13)	
Time		Pre (T1)	Post (T2)	Pre (T1)	Post (T2)
Dependent Variables Mean (SD)	Depressive Symptom	26.2 (10.3)	17.1 (9.49)	31.9 (11.5)	28.5 (9.97)
	Negative Affect	28.3 (6.49)	22.5 (5.94)	31.9 (9.7)	28.8 (3.15)
	Perceived Stress	25.1 (4.21)	17.5 (3.82)	25.1 (3.84)	23.5 (3.15)
	Ruminative thoughts	55.9 (14.8)	45.2 (12.52)	63.8 (14.5)	59.1 (14.64)
	Positive Affect	28.1 (5.35)	34.1 (5.96)	28.6 (3.84)	30.8 (6.67)
	Resiliency	25.7 (6.32)	26.2 (4.04)	23.3 (7.64)	22 (6.66)
Covariate Mean (SD)	Nature-Relatedness (Total)	23.9 (3.59)	N/A	21.8 (3.39)	N/A

**Table 3:** Estimated marginal means plot of the interaction between time and condition for all six dependent variables.

Depressive Symptoms	* 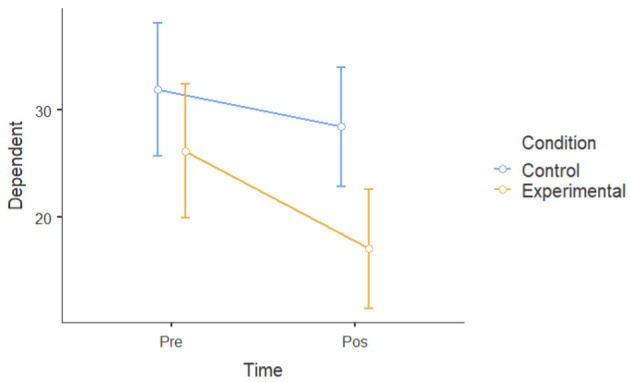 *
Negative Affect	* 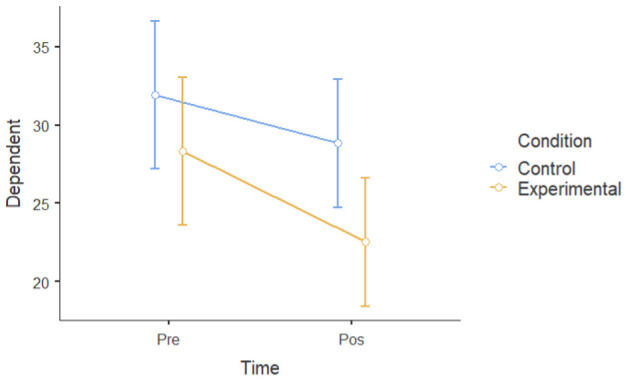 *
Perceived Stress	* 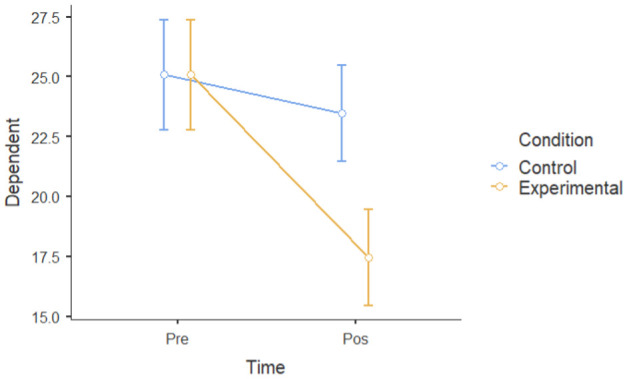 *
Ruminative Thoughts	* 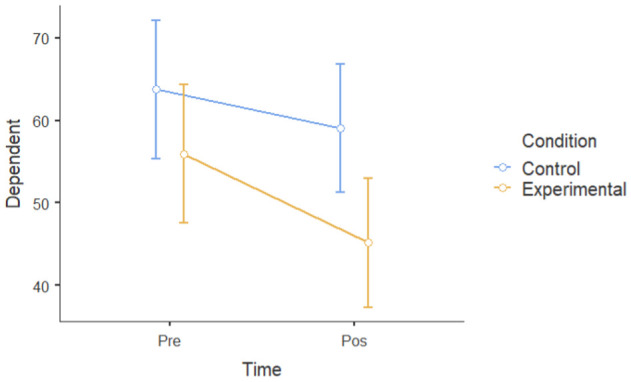 *
Positive Affect	* 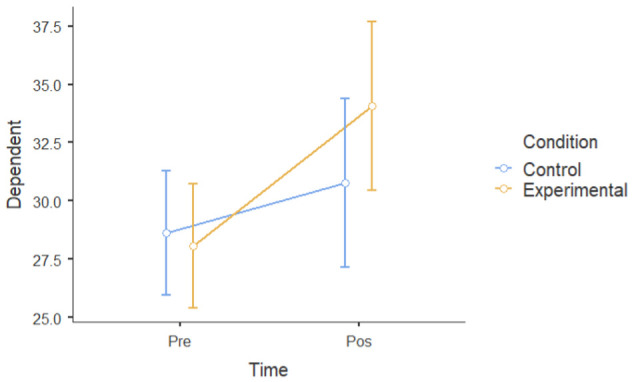 *
Resilience Scores	* 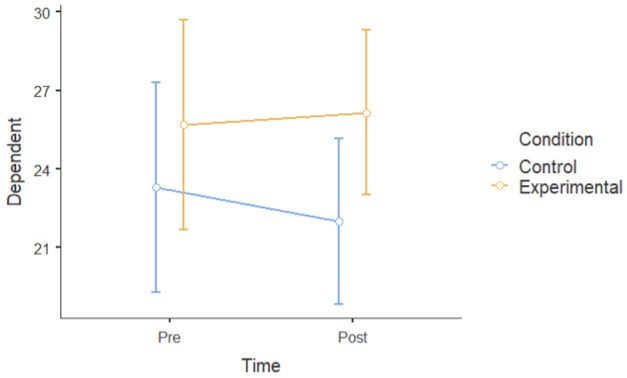 *

**Table 4:** Scatterplots of the interaction between nature-relatedness and all six dependent variables at post-intervention.

Depressive Symptoms	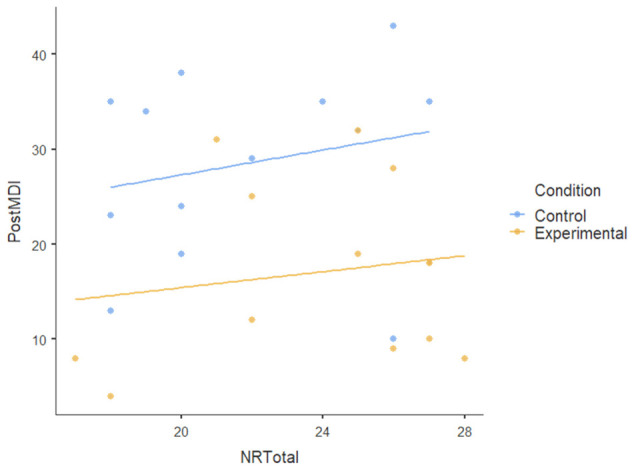
Negative Affect	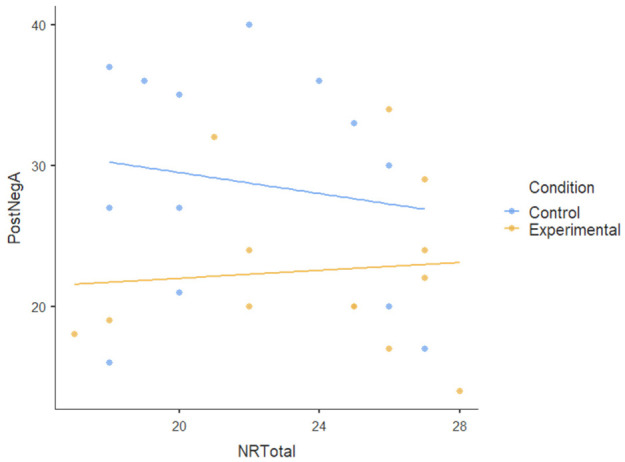
Perceived Stress	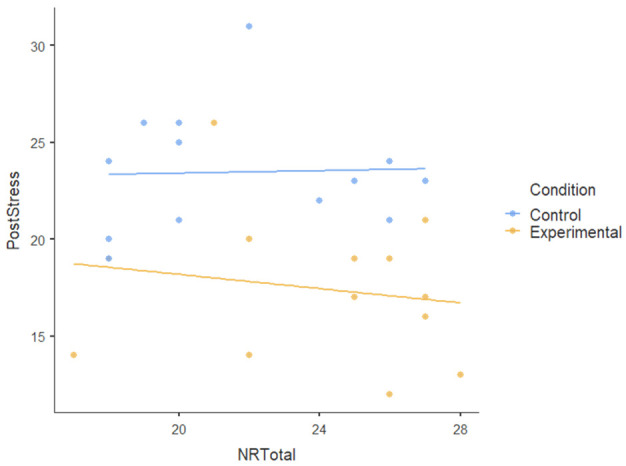
Ruminative Thoughts	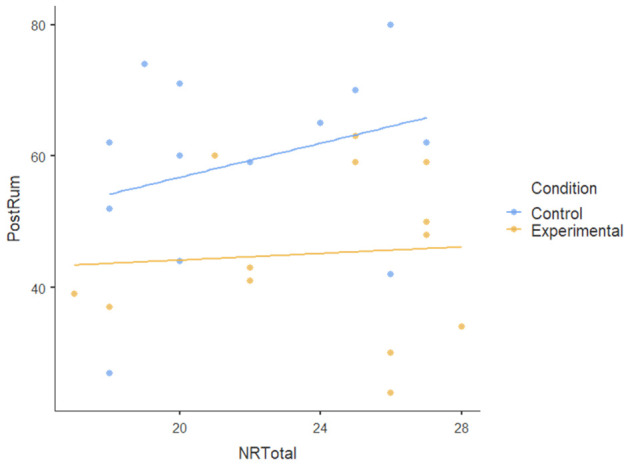
Positive Affect	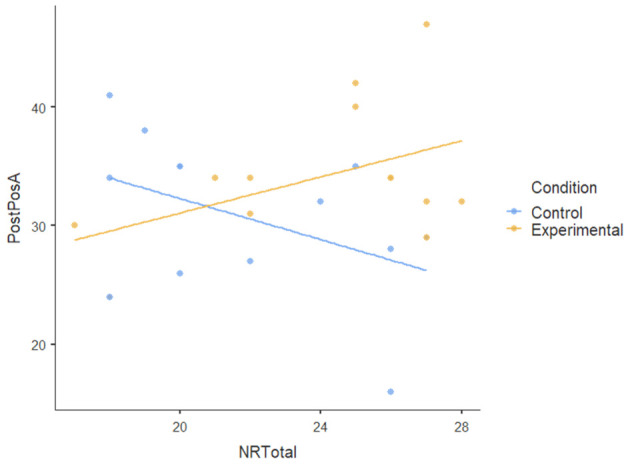
Resilience Scores	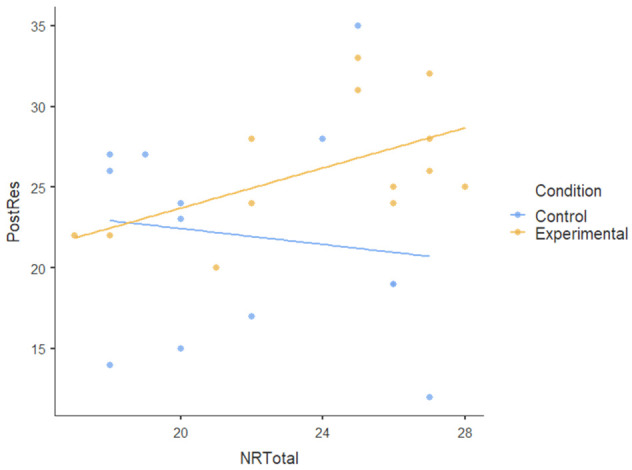

**Table 5:** Experiential and conceptual themes with descriptions of participants’ experiences tending to indoor plants.

Themes/Subthemes	Description
In Common: A first step but not the only stop	Participants differ on the impact, i.e., tending to indoor plants has caveats and recognising this nature-based intervention is not a complete or standalone solution to coping for all
**1. Offers a slice of nature by bringing the outside in**	Captures how indoor plants provide us with the natural world in the comfort of our homes in our everyday lives
Build connection with the larger natural world	Forming a connection with the larger natural world, offering the psychological and emotional benefits associated that may have otherwise been missed
First steps in building a bridge to nature.	Reports that indoor plant intervention encouraged participants to take the first step to building a bridge with nature
**2. Fosters an emotionally regulating personal sanctuary**	Captures how indoor plants foster a structured sanctuary, providing a personal retreat where participants can build structure, reflect, and find relief and emotional stability that helps mitigate the tendency to ruminate
Positively enhancing moods	Participants consistently described how tending to plants had positively enhanced their mood (sanctuary’s positive benefits)
A structured coping mechanism via distractions	Indoor plants are a distraction, focusing participants’ attention away from negative repetitive thoughts (sanctuary’s ways)
Sense of empowerment and agency	The sense of control and responsibility participants experience while tending to the plant (sanctuary’s ways)
Momentary constant respite	Tending to indoor plants offers participants a consistent break, i.e., constant in their life that provides relaxation and relief (sanctuary’s ways)
**3. Plants seeds for improving self-care, personal growth, and introspection**	Participants address the relationship between resilience and indoor plants; the lessons learned by tending to plants taught participants about personal growth and overcoming challenges
Symbolic representation of renewal	Participants consider indoor plants as a symbolic representation of renewal and forward thinking; they see their own personal growth reflected in their care for plants
Mirroring psychological resilience	Participants realise that, similar to indoor plants, we also endure neglect and thrive through challenges, and that tending to a plant serves as a metaphor for self-care and personal growth, helping the individual build resilience by validating their ability to nurture life, thus mirroring psychological resilience

## Data Availability

Data supporting reported results can be obtained upon request via Microsoft Excel. No raw data have been currently archived.

## Supplementary Materials

The following supporting information can be downloaded at: https://www.mdpi.com/article/10.3390/ijerph22030369/s1, Supplementary S1: Ethical Approval; Supplementary S2: List of Quantitative Measures asked in Qualtrics; Supplementary S3: Interview Schedule; Supplementary S4: Example of coded interview transcript (001); Supplementary S5: Photo of ZZ plants how-to-care note (experimental group); Supplementary S6: Few Pages of Self-Help Pamphlet (control group); Supplementary S7: QQ plots of dependent variables (normality assumptions); Supplementary S8: Personal Reflections on the Research Process.
