# Supplementary material for: Thriving Through Stressful Life Events with Nature: A Mixed-Method Study on Tending Indoor Plants and Rumination Resilience
Source: Int J Environ Res Public Health. 2025 Mar 3;22(3):369. doi: 10.3390/ijerph22030369 (PMC11942186; doi:10.3390/ijerph22030369)
Supplement: Supplementary file 1 [file ijerph-22-00369-s001.zip › ijerph-3434734-supplementary.pdf]

Supplementary Materials

Supplementary S1: Ethical Approval

RESEARCH & INNOVATION SERVICES
SURREY.AC.UK/RIS

UNIVERSITY OF SURREY

Research Integrity & Governance Office (RIGO)
4th floor Senate House
Guildford, Surrey GU2 7XH UK

T: +44 (0)1483 68 9103/2051

E: ethics@surrey.ac.uk
W: www.surrey.ac.uk

Samieul Azad
Psychology
Faculty of Health and Medical Sciences

25 June 2024

Dear Samieul,

EGA ref: FHMS 23-24 160 EGA
Project Title: Thriving through Stressful Life Events with Nature: A mixed method research on tending indoor plants and rumination resilience

On behalf of the University Ethics Committee (UEC), I am pleased to confirm a 'Favourable Ethical Opinion' (FEO) for the above research on the basis of the submitted protocol and final supporting documentation listed in the table below.

Date of confirmation of ethical opinion: 25th June 2024.

This opinion is given on the understanding that you will comply with the relevant University policies, ethical and professional standards and any applicable regulatory requirements, and have completed all mandatory training provided by the University of Surrey.

The final list of documents reviewed by the Committee is as follows:

Document	Version	Date
SAGE-HDR Form - 6837020 (1)	1046015-1045997-123575935	15/04/2024
EGA Study Protocol – 6837020	2.0	30/05/2024
RIGO Ethics and Governance Application Form - 6837020	2.0	30/05/2024
Baseline Consent Form - 6837020	2.1	30/05/2024
Data Management Plan - 6837020	2.0	30/05/2024
Debrief Form - 6837020	2.0	30/05/2024
Interview Consent Form – 6837020	2.1	30/05/2024
Measures Used – 6837020	1.0	30/05/2024
Participant Information Sheet – 6837020	2.0	30/05/2024
Team Summary – 6837020	1.0	30/05/2024
Self-Help Pamphlet	-	15/04/2024
FHMS 23-24 160 EGA Amendments_6837020_30 May 2024 – researcher's response	-	30/05/2024

Yours sincerely,

Dr Hana Hassanin
Chair of the University Ethics Committee
RIGO: JN

Copy to: Melissa Marselle

Supplementary S2: List of Quantitative Measures asked in Qualtrics

Inclusion Criteria: Stressful Life Event Data

1) List of Threatening Experiences Questionnaire (LTE-Q) (Brugha & Cragg, 1990)

Please read each of the items below and indicate yes or no with an x, if you have experienced these in the last six months.

Table 1 Original version of the List of Threatening Events Questionnaire (LTE-Q) developed by Brugha et al.[17]

Item	Question	Yes	No
1	You yourself suffered a serious illness, injury, or an assault		
2	A serious illness, injury, or assault happened to a close relative		
3	Your parent, child, or spouse died		
4	A close family friend or another relative (aunt, cousin, grandparent) died		
5	You had a separation due to marital difficulties		
6	You broke off a steady relationship		
7	You had a serious problem with a close friend, neighbor, or relative		
8	You became unemployed or you were seeking work unsuccessfully for more than one month		
9	You were sacked from your job		
10	You had a major financial crisis		
11	You had problems with the police and a court appearance		
12	Something you valued was lost or stolen		

Covariate Data:

1) Demographic Information (Age, Sex, Country/City, Occupation)

2) Nature Relatedness Scale (NR-6, (Nisbet and Zalenski,2013)

Instructions: For each of the following, please rate the extent to which you agree with each statement, using the scale from 1 to 5 as shown below. Please respond as you really feel, rather than how you think "most people" feel.

1	2	3	4	5
Disagree strongly	Disagree a little	Neither agree or disagree	Agree a little	Agree strongly

a. My ideal vacation spot would be a remote, wilderness area.
b. I always think about how my actions affect the environment.
c. My connection to nature and the environment is a part of my spirituality.
d. I take notice of wildlife wherever I am.
e. My relationship to nature is an important part of who I am.
f. I feel very connected to all living things and the earth. (Nisbet et al, 2009)

Psychological/Mental Health Outcome:

1) Connor-Davidson Resilience Scale (CD-RISC-10)(Campbell-Sills & Seins, 2007)

Please read each of these items below and indicate whether you've felt these over the last month: not true at all(0), rarely true (1), sometimes true (2), often true (3),and true nearly all of the time (4).

1. Able to adapt to change
2. Can deal with whatever comes
3. Tries to see humorous side of problems
4. Coping with stress can strengthen me
5. Tend to bounce back after illness or hardship
6. Can achieve goals despite obstacles
7. Can stay focused under pressure
8. Not easily discouraged by failure
9. Thinks of self as strong person
10. Can handle unpleasant feelings

2) Major Depression Inventory, (Olson et al, 2004)

**How much of the time in the last month…**	All the time	Most of the time	Slightly more than half the time	Slightly less than half the time	Some of the time	At no time
1. Have you felt low in spirits or sad?	5 ☐	4 ☐	3 ☐	2 ☐	1 ☐	0 ☐
2. Have you lost interest in your daily activities?	5 ☐	4 ☐	3 ☐	2 ☐	1 ☐	0 ☐
3. Have you felt lacking in energy and strength?	5 ☐	4 ☐	3 ☐	2 ☐	1 ☐	0 ☐
4. Have you felt less self-confident?	5 ☐	4 ☐	3 ☐	2 ☐	1 ☐	0 ☐
5. Have you had a bad conscience or feelings of guilt?	5 ☐	4 ☐	3 ☐	2 ☐	1 ☐	0 ☐
6. Have you felt that life wasn´t worth living?	5 ☐	4 ☐	3 ☐	2 ☐	1 ☐	0 ☐
7. Have you had difficulty in concentrat-ing, e.g. when reading the newspaper or watching television?	5 ☐	4 ☐	3 ☐	2 ☐	1 ☐	0 ☐
8 a. Have you felt very restless?	5 ☐	4 ☐	3 ☐	2 ☐	1 ☐	0 ☐
8 b. Have you felt subdued or slowed down?	5 ☐	4 ☐	3 ☐	2 ☐	1 ☐	0 ☐
9 a. Have you slept too little?	5 ☐	4 ☐	3 ☐	2 ☐	1 ☐	0 ☐
9 b. Have you slept too much?	5 ☐	4 ☐	3 ☐	2 ☐	1 ☐	0 ☐
10 a. Have you suffered from reduced appetite?	5 ☐	4 ☐	3 ☐	2 ☐	1 ☐	0 ☐
10 b. Have you suffered from increased appetite?	5 ☐	4 ☐	3 ☐	2 ☐	1 ☐	0 ☐

3) Positive and Negative Affect Schedule (PANAS-SF) (Watson et al, 1988)

Positive and Negative Affect Schedule (PANAS-SF)

Indicate the extent you have felt this way over the past month		Very slightly or not at all	A little	Moderately	Quite a bit	Extremely
PANAS 1	Interested	☐ 1	☐ 2	☐ 3	☐ 4	☐ 5
PANAS 2	Distressed	☐ 1	☐ 2	☐ 3	☐ 4	☐ 5
PANAS 3	Excited	☐ 1	☐ 2	☐ 3	☐ 4	☐ 5
PANAS 4	Upset	☐ 1	☐ 2	☐ 3	☐ 4	☐ 5
PANAS 5	Strong	☐ 1	☐ 2	☐ 3	☐ 4	☐ 5
PANAS 6	Guilty	☐ 1	☐ 2	☐ 3	☐ 4	☐ 5
PANAS 7	Scared	☐ 1	☐ 2	☐ 3	☐ 4	☐ 5
PANAS 8	Hostile	☐ 1	☐ 2	☐ 3	☐ 4	☐ 5
PANAS 9	Enthusiastic	☐ 1	☐ 2	☐ 3	☐ 4	☐ 5
PANAS 10	Proud	☐ 1	☐ 2	☐ 3	☐ 4	☐ 5
PANAS 11	Irritable	☐ 1	☐ 2	☐ 3	☐ 4	☐ 5
PANAS 12	Alert	☐ 1	☐ 2	☐ 3	☐ 4	☐ 5
PANAS 13	Ashamed	☐ 1	☐ 2	☐ 3	☐ 4	☐ 5
PANAS 14	Inspired	☐ 1	☐ 2	☐ 3	☐ 4	☐ 5
PANAS 15	Nervous	☐ 1	☐ 2	☐ 3	☐ 4	☐ 5
PANAS 16	Determined	☐ 1	☐ 2	☐ 3	☐ 4	☐ 5
PANAS 17	Attentive	☐ 1	☐ 2	☐ 3	☐ 4	☐ 5
PANAS 18	Jittery	☐ 1	☐ 2	☐ 3	☐ 4	☐ 5
PANAS 19	Active	☐ 1	☐ 2	☐ 3	☐ 4	☐ 5
PANAS 20	Afraid	☐ 1	☐ 2	☐ 3	☐ 4	☐ 5

4) Perceived Stress Scale (PSS-10) (Cohen et al, 1983)

Sr No.	During last one month, how often you thought or felt a certain way	Never	Almost never	Sometimes	Fairly often	Very often
1.	How often have you been upset because of something that happened unexpectedly?	0	1	2	3	4
2.	How often have you felt that you were unable to control the important things in your life?	0	1	2	3	4
3.	How often have you felt nervous and "stressed"?	0	1	2	3	4
4.	How often have you felt confident about your ability to handle your personal problems?	0	1	2	3	4
5.	How often have you felt that things were going your way?	0	1	2	3	4
6.	How often have you found that you could not cope with all the things that you had to do?	0	1	2	3	4
7.	How often have you been able to control irritations in your life?	0	1	2	3	4
8.	How often have you felt that you were on top of things?	0	1	2	3	4
9.	How often have you been angered because of things that were outside of your control?	0	1	2	3	4
10.	How often have you felt difficulties were piling up so high that you could not overcome them?	0	1	2	3	4

Courtesy: Cohen S. "Perceived Stress Scale." Psychology. 1994:1–3.

5) Ruminative Response Scale (RRS) (Nolen-Hoeksema & Morrow, 1991, Treynor et al,2003)

Please read each of the items below and indicate whether you almost never, sometimes, often, or almost always think or do each one when you feel down, sad, or depressed. Please indicate what you generally do, not what you think you should do.
1 almost never 2 sometimes 3 often 4 almost always

1. think about how alone you feel
2. think "I won't be able to do my job if I don't snap out of this"
3. think about your feelings of fatigue and achiness
4. think about how hard it is to concentrate
5. think "What am I doing to deserve this?"
6. think about how passive and unmotivated you feel.
7. analyse recent events to try to understand why you are depressed
8. think about how you don't seem to feel anything anymore
9. think "Why can't I get going?"
10. think "Why do I always react this way?"
11. go away by yourself and think about why you feel this way
12. write down what you are thinking about and analyze it
13. think about a recent situation, wishing it had gone better
14. think "I won't be able to concentrate if I keep feeling this way."
15. think "Why do I have problems other people don't have?"
16. think "Why can't I handle things better?"
17. think about how sad you feel.
18. think about all your shortcomings, failings, faults, mistakes
19. think about how you don't feel up to doing anything
20. analyse your personality to try to understand why you are depressed
21. go someplace alone to think about your feelings
22. think about how angry you are with yourself

Supplementary S3: Interview Schedule

Did you tend to the plant? How often? What did that look like?
How is the indoor plant currently?
How did you feel while tending to the indoor plant? (Can you describe the emotions and thoughts that arise?)
Have you noticed any changes in your mood, or well-being while tending to the plant compared to before the intervention?
If you have experienced a time when your mind gets stuck on repetitive thoughts, how do you think tending to the plant affected the way you managed those thoughts?
Have there been any moments where you felt particularly connected to the natural environment outside while tending to the plant?
How do you envision continuing to tend indoor plants as part of your journey through your ruminative thoughts? /after going through a stressful life event?
What do you think about the relationship between resilience and tending to the indoor plant?

Supplementary S4: Example of coded interview transcript (001)

SA: Alright, hi so first question is. How is the indoor plant doing currently?	
001: it's good. It's still like alive and stuff. I think it looks OK. All, green. Yeah. No, no, brown, alright.	
SA: So what did tending the plant look like during the intervention?	
001: Well, I was sort of just like, I'll keep it obviously in the sun and then like, I'd feel it like every two to three days if it was dry and I would water it a little bit. But if it wasn't, I would usually just leave it 'cause when I would water, I'd water a lot. So it was fine for, like, a couple days at a time. I wasn't being like silly. I'm like absolutely drenching it. Like I was just covering. Like when I did it like I wouldn't, like, put it under the tap. Like I would put the water in my hands and then I'd like to put it in the soil through that way so that I can make sure that, like, the whole surface was wet. And then like, once a hole off the top was wet, that would, like, be enough.	
SA: OK, cool. Yeah. And then so how did you feel while tending to the park? Can you describe any emotions or thoughts that arose?	
001: I'm not going to lie, I didn't feel like Anything in particular I guess I always felt like a little worried when I watered it. Like, you know, is it OK? Like, is it gonna start dying soon or anything like that? And it was like, nice to like, take a couple minutes out of the day just to focus on, like, one small, like, a little, a little bit calming of a task, but nothing like particularly emotional or anything like that.	Didn't feel a big emotions or particular way<br><br>A little worry, to keep it alive<br><br>Felt calming to take time out the day to focus on something
SA: Why do you think it's nice to sort of take that time out the day?	
001: I don't know. It was just like. Time out of the day to focus on one task that was like stress free in like every capacity. It's like there was no like pressure like there was a slight pressure like in a sense like I didn't want the plant to die, but there was no pressure in like if I don't do this task it like I'm gonna get a bad grade or something like that. You know, like it was just a little thing to do every day. That was like Nice. And I was like caring for something. So it was just it was pretty relaxing. It was almost like. I would have equated it a little bit to like self-care or something like that like it felt like that sort of feeling. So self-care like when you don't know, put cream on your face or something like that.	Indoor plants help focus on task that stress free in every way/<br><br>Indoor plants don't add pressure, only slight to ensure its alive.<br><br>Not the end if they don't tend to plant, no negative feedback<br><br>Little thing to do every day, enjoyed caring for living being<br><br>Felt relaxing, equated to self-care
SA: Yeah. And then so, have you noticed any changes in your mood or well-being well, while	

tending to the plant compared to before the intervention?	
001: Long term, not really. I think it's pretty much the same. I think like I said in those little moments like it felt nice to just put a bit of my attention on the plant, but I didn't. I wouldn't say that like long term, I've noticed anything major. I was thinking like, oh, like it might might, like, feel like really connected to this plant. And like, you know, in my day-to-day, think about it a lot or I don't know something like that or like be happier. But it wasn't. It's just like and now, like, they don't really feel anything over it. It was just a plant, you know.	No mood change long term<br><br>Nice to have been given the little moment, to pull attention to caring<br><br>Desire to feel connected to the plant, or happier, but disappointed it never<br><br>Managed their expectations
SA: Uh huh. And then if you have experience with time where your mind got stuck kind of repetitive thoughts, how do you think tending to the plant affected the way you manage those thoughts?	
001: I guess maybe going back to the relaxation aspect of it like it was. Whenever I took the time to take care of the plant it made me like stop and think and, like, do something straightforward. So I guess that same thinking could be applied for like getting stuck on repetitive thinking. So you just like take a minute to stop and think and chill out. And then like, think of where you're going to go next from there. Although like I don't see any like. Any major emotional changes like that I do sort of see like you bringing that question up like there is a little bit of like a learning to just take a moment to step back and look at things.	Plant helps feel relaxed<br><br>Helped to stop and think, something straightforward<br><br>allowing for a break from repetitive thinking and providing a moment to step back and reflect.<br><br>development of a habit or skill in taking a moment to pause and consider one's next step
SA: Do you think it's difficult to learn and take a step back.	
001: Yeah definitely, Especially like. If you don't have anything that you're like a plant like you're watering on the daily and that's like a daily bit of time. You just take out of your day to just like, sit and like, not do nothing but, like, do something like relaxed. If you don't have something like that, then it's like, how else do you really learn those behaviours, you know, because there's a lot of, like, life moves really fast and you're always doing something or even if you're just, like sitting and watching TV, you're always watching something like, your mind's always like hyperactive. So I guess that's a good way to learn those emotions.it helps you to slow down a little bit.	Difficult to learn to take step back if you don't have a plant or anything to help you realise<br><br>Necessary to take do something relaxing out of your life demands.<br><br>use of simple, repetitive tasks to help manage mental wellness by providing a break from constant stimulation<br><br>importance of slowing down in a fast-paced life and how daily plant care encourages take reflective pace
SA: And then I said, been any particular moments where you felt connected to the natural environment outside while tending to the plant?	

001: Unfortunately, I wouldn't say so. Would have been nice to experience something like that, but I don't think like I felt like that at a particular point, no. I'm not too sure, to be honest what that would look like. Maybe just like. Yeah, yeah, I'm not really sure. I think it's one of those things where if I felt it, I would know. But I just didn't feel like it at all, but I also. In the same way as can't like to describe to you what it would be like exactly.	Didn't feel connected in particular during the intervention, despite desire<br><br>Emotional awareness<br><br>Unsure what that connection might look, you know when you know, uncertainty vs desire
SA: That's fair. And then how do you envision continuing to tend to the indoor plant as part of your journey through any ruminated thoughts or after going through a stressful life event?	
001: Probably in the same way. So I think like if you were to go through like a difficult life event having. Something consistent in your schedule would help, like watering the plant daily or every couple of days. So like, even if you don't feel like a particular emotional connection to it, just the fact that it's something in your schedule that you can stick to I think would be really helpful. So I would definitely continue to do that.	Continue to tend to the plant<br><br>importance of having a consistent task or routine<br><br>routine activities as a coping mechanism to manage stress or emotional challenge
SA: OK. And then sort of like a final question, what do you think about the relationship between resilient and tending to the indoor plants?	
001: I think it's quite strong actually. I think it's like if you don't have it, it's a good way to develop it. And if you already have it, it's a good way to act as a stepping stone because while like I, personally didn't experience any strong emotional connection, I did see it. I was like a useful tool to be used like as one a person had a desire to because again, like the repetition, just taking time out of your day to focus on something. Those are like the foundations. Of building resilience, I think. Yeah, even though I didn't like to observe it myself, like, I can definitely see. It that it could be used that way and I think I think that was just kind of an outlier and I'm expecting like all the other participants to probably have experienced it because all like. I'm not sure they were, but like all of the parts were there for it to like to exist, it just didn't for me, but I think it would be very useful regardless.	Strong relationship between resilience and tending to indoor<br><br>Good way to develop resilience and also use as stepping stone to develop more<br><br>Despite not seeing the connection, still consider tending to the plant a useful tool that helps focus on something<br><br>Use as a foundation<br><br>Recognizing the utility of routine activities as tools that can help develop emotional strength and resilience over time.<br><br>belief that other participants might experience this, individuals are different, have different experience, but the tool is useful regardless, however big the outcome
SA: And then yeah, that's all the questions I have. And then I'm wondering if you had any sort of final comments or anything you'd like to talk about the intervention go for it?	

001: Not particularly. Didn't really get my plant epiphany so. Yeah, I was hoping for it, but it didn't happen. But that's OK. It's just nice to have a plant.	
SA: Do you know what the plant epiphany would have been like? Because I don't know.	
001: I don't know either. Maybe I'm hinging too large of a moment on it.	
SA: Maybe, but I think it is good that you have that want for an epiphany even though it seems like such a strange huge moment that it. It seems I don't know how suddenly attainable it can be, but it's nice that you know you know you want it to happen even if we may never know when it happens.	
001: That is kind of true. Yeah. Yeah, I definitely do. So that's why I'll keep, like taking care of it. And I think maybe it's just a thing where you need, like, a longer space of time because something like that, like, it doesn't just happen overnight, you know. And even though this has been like a couple months, I think maybe like a year or so. It just needs time.	Continuing to tend to the plants, in hopes of develop a plant epiphany, however that may look like,<br><br>Know it may take more than a month or just one plant
SA: Yeah, yeah. And it is. It is just one plant as well. I'd say, I think.	
001: That is true. The more you have like, the bigger that environment sort of becomes. but I did definitely think like it's a first step. Like even just going for that one part. It's maybe like interested in getting other plants or maybe like taking care of it more. So it's definitely a good stepping stone. I feel like at the minimum.	Acknowledging this plant intervention may be the first step, want more plants and care for it long.<br><br>At the least it is a good stepping stone.
SA: Oh yeah, so did you have any questions for me or any queries you want to answer before we wrap things up	
001: No, I'm all good, to be honest. Although I would be really interested in reading your dissertation once it's finished.	
SA: Oh Yes, once it all completed. I'll be sure to send it over, Thank you for taking part.	

Supplementary S5: Photo of ZZ plants how-to-care note (experimental group)

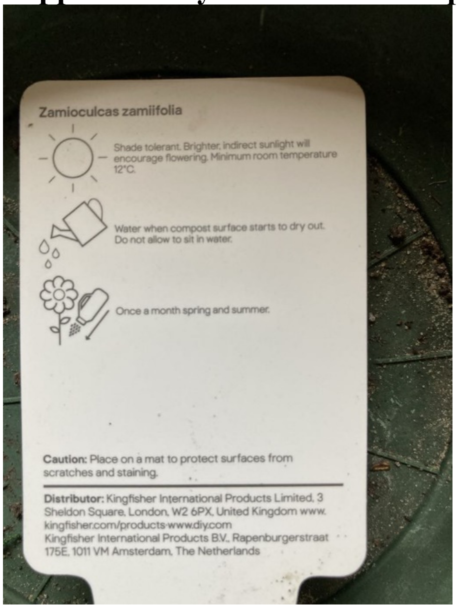

Supplementary S6: Few Pages of Self-Help Pamphlet (control group)

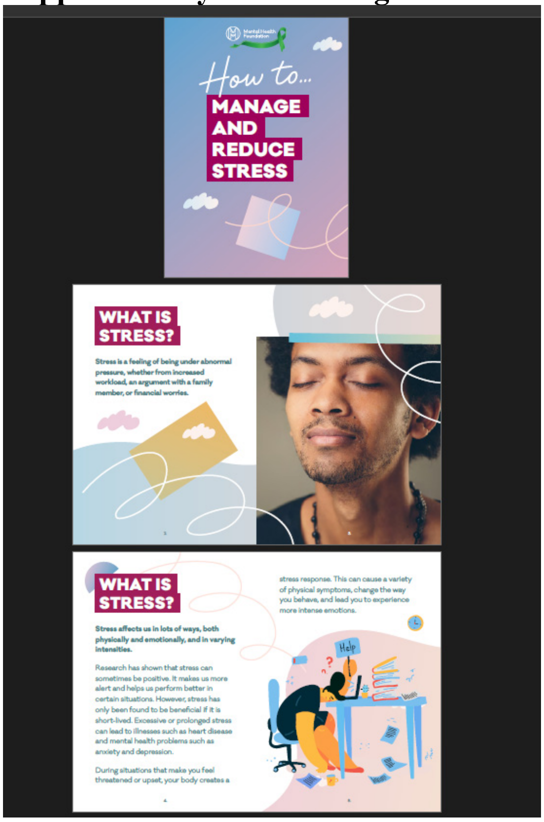

Supplementary S7: QQ plots of dependent variables (normality assumptions)

<table>
<tr><td>Depressive Symptom Post Intervention - PostMDI</td><td>Negative Affect Post Intervention - PostNegA</td></tr>
<tr><td>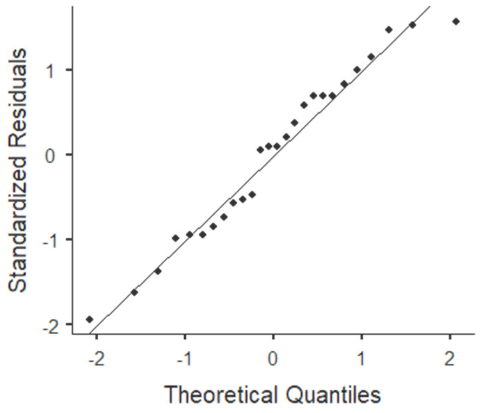
</td><td>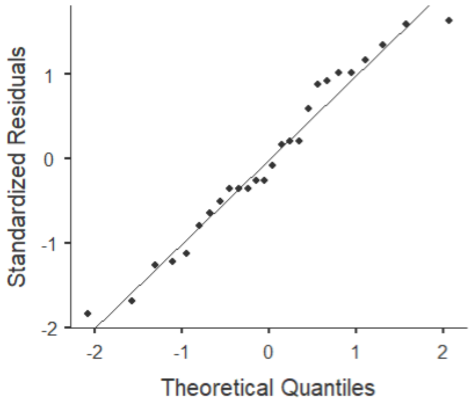
</td></tr>
</table>

<table>
<tr><td>Perceived Stress Post Intervention - PostStress</td><td>Ruminative thoughts Post Intervention - PostRum</td></tr>
<tr><td>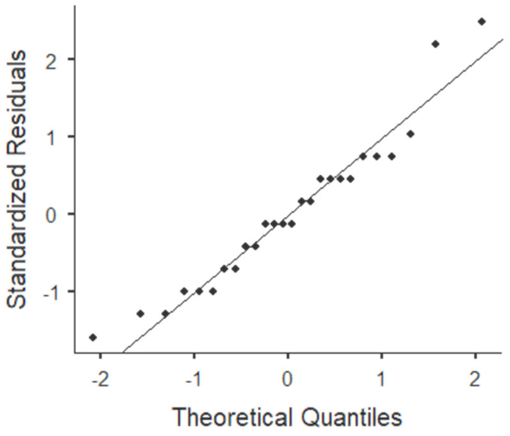
</td><td>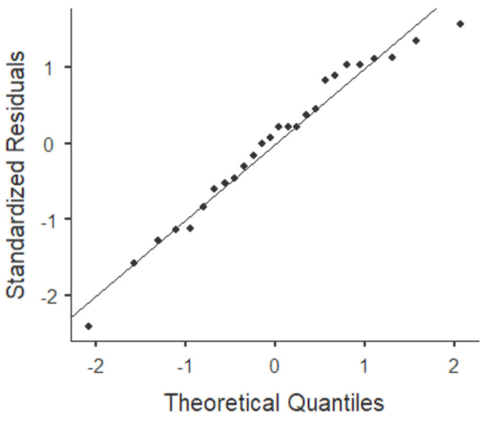
</td></tr>
</table>

Positive Affect Post Intervention - PostPosA	Resilience Score Post Intervention - PostRes

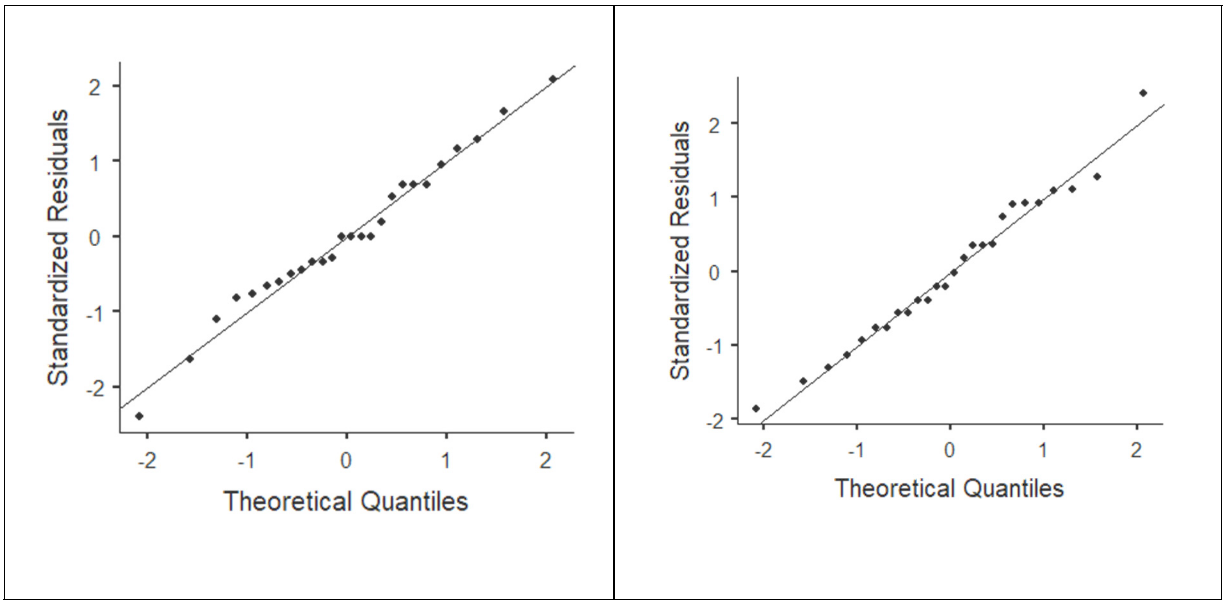

Standardized Residuals
2
1
0
-1
-2
-2
-1
0
1
2
Theoretical Quantiles
Standardized Residuals
2
1
0
-1
-2
-2
-1
0
1
2
Theoretical Quantiles

Supplementary S8: Personal Reflections on the Research Process

Upon reflecting on my personal investment on the research, I ensured that participants felt comfortable and that it was entirely up to them if they wanted to share their stressful life events in relation to the one-month nature intervention. The interview schedule avoided making participants bring up the event, although many did, it may have been to the detriment of the findings as there was no point of comparison between experiences (stressful life events vs tending to indoor plant). I think it was important I showed no affinity for nature and belief intending to indoor plants psychological benefits, so participants didn't feel incline to exaggerate their experience with tending to the indoor plant in order to meet my expectations. Also because I was the one personally delivering the plants and had financially invested into the nature-based intervention. A difficulty noted was time management regarding participant recruitment and scheduling interviews which made the analytic process a little less than smooth. Nevertheless, I think it was surprising but also wholesome to hear participants experiences on how profoundly the simplicity of tending to an indoor plant positively affected their everyday life. It was also recognising the diversity but also similarity of individual experiences given their challenges. I know how much this research had a positive impact on both me and the participants and its strange to think there are now 13 plants, all thriving in different homes, different lives because of this research.
